# Plasma fat-soluble vitamin and carotenoid concentrations after plant sterol and plant stanol consumption: a meta-analysis of randomized controlled trials

**DOI:** 10.1007/s00394-016-1289-7

**Published:** 2016-09-03

**Authors:** Sabine Baumgartner, Rouyanne T. Ras, Elke A. Trautwein, Ronald P. Mensink, Jogchum Plat

**Affiliations:** 1 Department of Human Biology, NUTRIM School of Nutrition and Translational Research in Metabolism, Maastricht University, PO Box 616, 6200 MD Maastricht, The Netherlands; 2 Unilever R&D Vlaardingen, Vlaardingen, The Netherlands

**Keywords:** Plant sterols, Plant stanols, Cholesterol, Hydrocarbon carotenoids, Oxygenated carotenoids, Fat-soluble vitamins

## Abstract

**Purpose:**

Plant sterols and stanols interfere with intestinal cholesterol absorption, and it has been questioned whether absorption and plasma concentrations of fat-soluble vitamins and carotenoids are also affected. We conducted a meta-analysis to assess the effects of plant sterol and stanol consumption on plasma fat-soluble vitamin and carotenoid concentrations.

**Methods:**

Forty-one randomized controlled trials involving 3306 subjects were included. Weighted absolute and relative changes of non-standardized and total cholesterol (TC)-standardized values (expressed as summary estimates and 95 % CIs) were calculated for three fat-soluble vitamins (α- and γ-tocopherol, retinol and vitamin D) and six carotenoids (β-carotene, α-carotene, lycopene, lutein, zeaxanthin and β-cryptoxanthin) using a random effects model. Heterogeneity was assessed using predefined subject and treatment characteristics.

**Results:**

Average plant sterol or stanol intake was 2.5 g/d. Relative non-standardized and TC-standardized concentrations of β-carotene decreased by, respectively, −16.3 % (95 % CI −18.3; −14.3) and −10.1 % (−12.3; −8.0), α-carotene by −14.4 % (−17.5; 11.3) and −7.8 % (−11.3; −4.3), and lycopene by −12.3 % (−14.6; −10.1) and −6.3 % (−8.6; −4.0). Lutein concentrations decreased by −7.4 % (−10.1; −4.8), while TC-standardized concentrations were not changed. For zeaxanthin, these values were −12.9 % (−18.9; −6.8) and −7.7 % (−13.8; −1.7) and for β-cryptoxanthin −10.6 % (−14.3; −6.9) and −4.8 % (−8.7; −0.9). Non-standardized α-tocopherol concentrations decreased by −7.1 % (−8.0; −6.2) and γ-tocopherol by −6.9 % (−9.8; −3.9), while TC-standardized tocopherol concentrations were not changed. Non-standardized retinol and vitamin D concentrations were not affected. Results were not affected by baseline concentrations, dose, duration and type of plant sterols/stanols, except for significant effects of duration (≤4 vs. >4 weeks) on TC-standardized lutein concentrations (1.0 vs. −5.6 %) and type of plant sterol/stanol on TC-standardized β-carotene concentrations (−8.9 vs. −14.2 %).

**Conclusions:**

Plant sterol and stanol intake lowers TC-standardized hydrocarbon carotenoid concentrations, differently affects TC-standardized oxygenated carotenoid concentrations, but does not affect TC-standardized tocopherol concentrations or absolute retinol and vitamin D concentrations. Observed concentrations remained within normal ranges.

**Electronic supplementary material:**

The online version of this article (doi:10.1007/s00394-016-1289-7) contains supplementary material, which is available to authorized users.

## Introduction

Numerous studies have shown that consuming plant sterol- or plant stanol-enriched foods (0.6–3.3 g sterols or stanols/day) lowers plasma or serum low-density lipoprotein cholesterol (LDL-C) concentrations by 6−12 % [1–3]. Since plant sterols and plant stanols interfere with intestinal cholesterol absorption and consequently affect whole-body lipid and lipoprotein metabolism [4], questions have been raised whether plasma fat-soluble vitamin and carotenoid concentrations are also affected by plant sterol and stanol consumption. A reduction in plasma fat-soluble vitamin and carotenoid concentrations may be undesirable, since in prospective cohort studies lower concentrations have been associated with an increased risk of several chronic diseases, such as cardiovascular diseases (CVDs), cancer and age-related macular degeneration (AMD) [5–7], although evidence from randomized controlled trials is lacking [8, 9].

Already in 2003, Katan et al. [10] performed a meta-analysis including 18 studies and showed significant reductions in circulating α-tocopherol, α-carotene, β-carotene and lycopene concentrations after plant sterol and stanol ester consumption. Concentrations of the fat-soluble vitamins retinol and vitamin D were not affected. However, when concentrations were standardized for serum total cholesterol (TC) concentrations, there were no longer decreases in α-tocopherol, α-carotene and lycopene concentrations, while β-carotene concentrations remained significantly reduced with a mean reduction of 12 %. This implies that at least for some of the vitamins and carotenoids decreases are related to the reduction in the number of circulating lipoproteins, the carriers of the fat-soluble tocopherols and carotenoids. However, it has also been suggested that plant sterols and stanols reduce the incorporation of the more lipophilic hydrocarbon carotenoids (i.e., β-carotene, α-carotene and lycopene) into the micelles to a greater extent than those of the less lipophilic oxygenated carotenoids (lutein, zeaxanthin and β-cryptoxanthin) and tocopherols [11]. Finally, plant sterol and plant stanol consumption might not affect vitamins that require a specific transporter instead of the lipoprotein-mediated transport route, such as retinol and vitamin D.

Recently, Fardet et al. [12] have summarized the effects of plant sterols and plant stanols on fat-soluble vitamin and carotenoid concentrations. They calculated median relative changes (standardized and non-standardized) in α-tocopherol, γ-tocopherol, α-carotene, β-carotene and β-cryptoxanthin concentrations. However, results seem biased as only studies were included that reported significant reductions in plasma fat-soluble vitamin and carotenoid concentrations. In addition, a detailed description of the systematic approach to review the available literature was lacking. Therefore, to provide an up-to-date quantitated overview on the effects of plant sterol or plant stanol consumption on plasma fat-soluble vitamin and carotenoid concentrations, we performed a meta-analysis of randomized controlled trials (RCTs). For this, absolute and relative changes were estimated for three fat-soluble vitamin (α- and γ-tocopherol, retinol and vitamin D) and six carotenoid (β-carotene, α-carotene, lycopene, lutein, zeaxanthin and β-cryptoxanthin) concentrations after plant sterol or plant stanol consumption. Furthermore, potential sources of heterogeneity between studies were assessed by investigating the impact of predefined subject and treatment characteristics on changes in plasma fat-soluble vitamin and carotenoid concentrations.

## Methods

### Search strategy

Potentially relevant studies were retrieved by a systematic search of five databases (Medline, Embase, the Cochrane Library, Cab abstracts and Food, Science and Technology abstracts) in December 2014. A search strategy was developed including the Medical Subject Heading ‘phytosterols’ and the following search terms: (plant sterol* or phytosterol* or sitosterol* or campesterol* or stigmasterol* or brassicasterol* or plant stanol* or sitostanol* or campestanol*) and (vitamin* or carotene* or carotenoid* or tocotrienol* or tocopherol* or alpha-carotene* or beta-carotene* or lycopene* or lutein* or zeaxanthin* or retinol* or calciferol*), limited to humans without any restriction on language. Throughout this paper, the term ‘plasma’ is used, irrespective whether plasma or serum concentrations were reported in the different studies.

### Selection of studies

Human intervention studies were considered eligible if effects of plant sterol or plant stanol consumption on plasma fat-soluble vitamin or carotenoid concentrations were reported. In the first selection round, titles and abstracts were screened and studies were selected if they met the following inclusion criteria: (1) RCT in humans (studies with children were allowed), (2) oral intake of plant sterol- or plant stanol-enriched foods or supplements, (3) measurement of plasma fat-soluble vitamins or carotenoids, (4) no co-intervention next to the plant sterol or plant stanol intervention, (5) no studies in phytosterolemic patients, (6) duration of at least 2 weeks and (7) no duplicates. For the second selection round, full publications were read to assess their eligibility. Studies were excluded when they lacked a control group or were not randomized, when an intentional co-intervention (e.g., extra vitamins) was given that could not be separated from the effects of the plant sterol or plant stanol intervention or when plasma fat-soluble vitamin or carotenoid concentrations were not reported. Studies evaluating the effects of ferulated plant sterols (as in shea nut oil) were not included, since they are not commonly used in currently commercially available plant sterol- and stanol-enriched foods and there is no consensus on their cholesterol-lowering effects [13, 14]. When inconclusive, the eligibility of studies was discussed among authors until consensus was reached.

### Data extraction and transformation

Data were collected using a database that included (1) publication characteristics (reference number, first author and year of publication), (2) study characteristics (parallel or crossover design, sample size and study duration), (3) subject characteristics (health status, mean age, mean BMI and gender distribution), (4) treatment characteristics (use of plant sterols or plant stanols, dose of plant sterols or plant stanols and food format), (5) measurement characteristics (serum or plasma) and (6) outcome variables [plasma and serum concentrations of α-carotene, β-carotene, α-tocopherol, γ-tocopherol, lutein, zeaxanthin, β-cryptoxanthin, lycopene, vitamin D, retinol, TC, LDL-C, high-density lipoprotein cholesterol (HDL-C) and triacylglycerols (TAG)].

For all outcome variables, mean concentrations and accompanying variance measures were extracted at baseline and at the end of the intervention. In the circulation, tocopherols and carotenoids are transported by lipoprotein particles and therefore often standardized for serum cholesterol concentrations. Since vitamin D and retinol are not transported by lipoproteins, they were solely expressed as non-standardized concentrations. To unify the use of all cholesterol-standardized tocopherol and carotenoid concentrations, absolute TC data were extracted and TC-standardized tocopherol and carotenoid concentrations were calculated by dividing the absolute tocopherol/carotenoid concentrations at baseline and at end of intervention by the respective TC concentrations. Original authors were contacted for non-standardized tocopherol and carotenoid concentrations if only cholesterol-standardized levels were reported [15–19].

Cholesterol data (TC, LDL-C and HDL-C) and TAG data expressed in mg/dL were converted into mmol/L by using their molecular weights (386.7 and 885.7 g/mol, respectively). In case fat-soluble vitamin or carotenoid concentrations were expressed in ng/mL, ng/dL, μg/mL, μg/dL, μg/L, mg/dL, mg/L, g/mL, these data were also transformed based on their molecular weights to derive concentrations in μmol/L or nmol/L (g/mol for α- and β-carotene: 536.9, α-tocopherol: 430.7, γ-tocopherol: 416.7, lutein and zeaxanthin: 568.9; β-cryptoxanthin: 552.9, lycopene: 536.9, retinol: 286.5 and vitamin D: 400.6). These transformations were done for means and for SEs or SDs.

Placebo-adjusted absolute and relative changes with accompanying SEs were calculated for α-carotene, β-carotene, α-tocopherol, γ-tocopherol, lutein, zeaxanthin, β-cryptoxanthin, lycopene, vitamin D, retinol, TC, LDL-C, HDL-C and TAG for each study (when data were available). For parallel studies, absolute and relative changes (with accompanying SEs) were calculated based on average concentrations and variance measures at baseline and at end of intervention of treatment and control groups. For crossover studies, absolute and relative changes were calculated based on concentrations at end of intervention of treatment and control periods. In case it was not possible to calculate placebo-adjusted changes and SEs, these data were used as reported in the papers [16, 20–25]. Data expressed as median (minimum, maximum) values were transformed to means ± SDs using the method of Wan et al. [26, 27] and data displayed in graphs were extracted using ScanIt version 1.0 [23, 28–31]. To derive SE from 95 % CI [13, 14] or from an effect estimate and *P* value [20], equations were used as described by the Cochrane handbook for systematic reviews of interventions [32]. The Friedewald equation [33] was used for one study [34] to estimate the TC concentration based on the reported concentrations of LDL-C, HDL-C and TG. In case only TC-standardized data were available, these data were added in SAS [23].

### Statistical analysis

For each outcome parameter, a weighted net effect (expressed as summary estimate and 95 % CI) was calculated using a random effects model and the inverse of the within-study variance (1/SE^2^) was used as weighing factor [35]. These effects were calculated for baseline concentrations, end-of-intervention concentrations, absolute and relative changes of non-standardized and TC-standardized values. For interpretation of the data, the various outcome parameters were clustered in hydrocarbon or oxygenated carotenoids, in fat-soluble vitamins or plasma lipids. Forest plots were made for relative changes in representatives of the three fat-soluble vitamin/carotenoid categories (β-carotene representing the hydrocarbon carotenoids, lutein the oxygenated carotenoids and α-tocopherol the tocopherols).

Funnel plots were created to visualize the likeliness of heterogeneity (in case many effect sizes fall outside the confidence limits) and publication bias (in case of asymmetry). Heterogeneity was assessed using Cochran’s *Q* test (*P* < 0.1 indicates significant heterogeneity) and quantified by I^2^, which indicates the percentage of variability in effect estimate that is due to heterogeneity rather than sampling error [35, 36]. Publication bias was evaluated by Egger’s weighted regression test where the absence of publication bias is reflected by an intercept close to 0 (*P* ≥ 0.05) [37].

Subgroup analyses were performed for β-carotene, lutein and α-tocopherol as representatives of the hydrocarbon carotenoids, oxygenated carotenoids and tocopherols to evaluate whether TC-standardized absolute and relative changes were influenced by predefined subject characteristics (i.e., baseline concentrations of β-carotene, lutein and α-tocopherol) or treatment characteristic (i.e., use of plant sterols or plant stanols, dose of plant sterols or plant stanols and duration of intervention). Subgroup analyses were also performed for effects on TC. Subgroups were defined using the median baseline concentrations and the median duration (4 weeks) as cut-offs. For the dose categories, we used 1.6, 2.0, 3.0 and 9.0 g/d as cut-offs allowing a more or less equal distribution of studies across the four dose categories.

Results were considered to be statistically significant if *P* < 0.05 based on two-sided testing. All statistical analyses were performed using SAS version 9.4 (SAS institute Inc., Cary, NC, USA). Forest plots were created using STATA version 12.1 (STATA Corporation, College Station, TX, USA). This meta-analysis adheres to the PRISMA statement guidelines for reporting in systematic reviews and meta-analyses.

## Results

### Overview of included studies

**Fig. 1:**
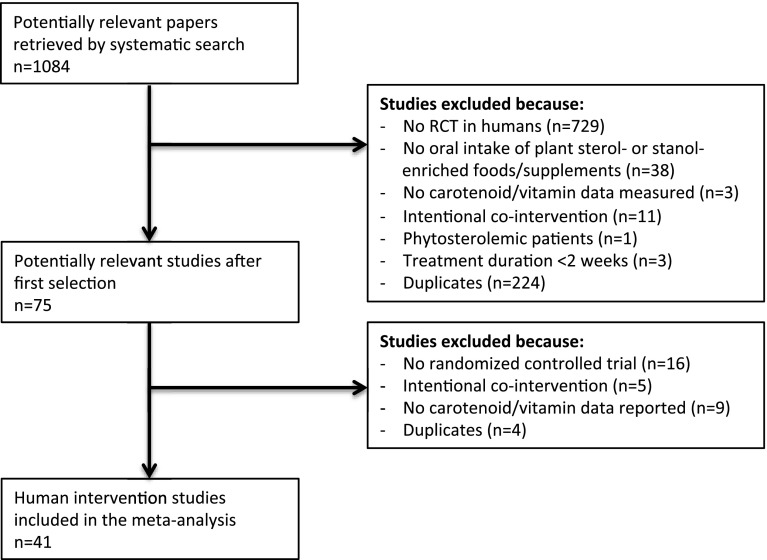
Flowchart study selection process. The literature search retrieves 1084 potentially relevant papers, 1009 are excluded after screening titles and abstracts, 75 articles are reviewed in full and 41 randomized controlled trials (RCTs) are included in the meta-analysis

The systematic search retrieved 1084 potentially relevant papers, and after two selection rounds, 41 RCTs were included in the meta-analysis. A flowchart of the study selection process is presented in Fig. 1.

Of the 41 included studies (Online Supplemental Material Tables 1 and 2), 23 were conducted as a parallel study [15–17, 19, 21–26, 28–31, 34, 38–45] and 18 studies had a crossover design [13, 14, 18, 20, 46–59]. In total, 3306 subjects participated with an average age of 47.6 years (range 10.5–61.8 years) and an average BMI of 25.1 kg/m^2^ (range 19.0–28.3 kg/m^2^). The median study duration was 28 days (range 21–364 days) with an average dose of 2.5 g/d (range 0.45–9.0 g/d), and 80 % of the studies were performed with esterified plant sterols or plant stanols.

### Plasma fat-soluble vitamin/carotenoid outcomes

The weighted effects of plant sterol or plant stanol consumption on plasma fat-soluble vitamin and carotenoid concentrations are presented in Table 1. Non-standardized and TC-standardized hydrocarbon carotenoid concentrations, i.e., lycopene, α-carotene and β-carotene, were significantly (*P* < 0.0001) lowered after consumption of plant sterol- or plant stanol-enriched foods. β-Carotene concentrations decreased on average by 0.08 µmol/L (−16.3 %; −10.1 % when TC-standardized), α-carotene concentrations by 0.02 µmol/L (−14.4 %; −7.8 % when TC-standardized) and lycopene concentrations by −0.04 µmol/L (−12.3 %; −6.3 % when TC-standardized). For the oxygenated carotenoids, i.e., lutein, zeaxanthin and β-cryptoxanthin, non-standardized concentrations were significantly (*P* < 0.0001) lowered, while changes in TC-standardized concentrations showed less conclusive results. Lutein concentrations decreased on average by 0.02 µmol/L (−7.4 %), while the TC-standardized concentrations in lutein were not significantly changed (−1.5 %) after plant sterol or plant stanol consumption. Non-standardized zeaxanthin concentrations decreased on average by 7.81 nmol/L (−12.9 %; −7.7 % when TC-standardized (*P* < 0.05)) and β-cryptoxanthin decreased by −0.03 µmol/L [−10.6 %; −4.8 % when TC-standardized (*P* < 0.05)]. Non-standardized tocopherol concentrations significantly (*P* < 0.0001) decreased after plant sterol or plant stanol consumption (α-tocopherol on average by 2.43 µmol/L (−7.1 %) and γ-tocopherol by 0.17 µmol/L (−6.9 %). However, when standardized for TC concentrations, α- and γ-tocopherol concentrations were no longer changed after plant sterol or plant stanol consumption (−0.3 and 0.2 %, respectively; *P* > 0.05). Concentrations of retinol and vitamin D were not changed after plant sterol or plant stanol consumption (−0.8 and 1.4 %, respectively; *P* > 0.05).

**Table 1:** Effects of plant sterol or plant stanol consumption on plasma carotenoid, fat-soluble vitamin, lipid and lipoprotein concentrations

	*n* (strata)	Unit	Baseline concentration^a^	Concentration after PS intervention^b^	Absolute change versus placebo	Relative change versus placebo
*Hydrocarbon carotenoids*						
β-Carotene	53	µmol/L	0.60 (0.54; 0.67)	0.52 (0.46; 0.57)	−0.08 (−0.09; −0.07)^1^	−16.3 (−18.3; −14.3)^1^
	54	µmol/mmolTC	0.10 (0.09; 0.11)	0.09 (0.08; 0.10)	−0.01 (−0.01; −0.01)^1^	−10.1 (−12.3; −8.0)^1^
α-Carotene	38	µmol/L	0.19 (0.15; 0.24)	0.18 (0.14; 0.22)	−0.02 (−0.02; −0.01)^1^	−14.4 (−17.5; −11.3)^1^
	37	µmol/mmolTC	0.03 (0.03; 0.04)	0.03 (0.02; 0.04)	0.00 (0.00; 0.00)^1^	−7.8 (−11.3; −4.3)^1^
Lycopene	42	µmol/L	0.57 (0.49; 0.65)	0.54 (0.45; 0.63)	−0.04 (−0.04; −0.03)^1^	−12.3 (−14.6; −10.1)^1^
	41	µmol/mmolTC	0.10 (0.08; 0.11)	0.10 (0.08; 0.11)	0.00 (−0.01; 0.00)^1^	−6.3 (−8.6; −4.0)^1^
*Oxygenated carotenoids*						
Lutein	23	µmol/L	0.38 (0.31; 0.45)	0.34 (0.29; 0.40)	−0.02 (−0.03; −0.01)^1^	−7.4 (−10.1; −4.8)^1^
	22	µmol/mmolTC	0.06 (0.05; 0.07)	0.06 (0.05; 0.07)	0.00 (0.00; 0.00)	−1.5 (−4.6; 1.6)
Zeaxanthin	13	nmol/L	63.90 (52.22; 75.57)	55.39 (45.13; 65.65)	−7.81 (−11.09; −4.53)^1^	−12.9 (−18.9; −6.8)^1^
	12	nmol/mmolTC	10.72 (8.34; 13.09)	9.78 (7.74; 11.81)	−0.70 (−1.20; −0.21)^2^	−7.7 (−13.8; −1.7)^3^
β-Cryptoxanthin	17	µmol/L	0.28 (0.22; 0.35)	0.26 (0.20; 0.32)	−0.03 (−0.04; −0.02)^1^	−10.6 (−14.3; −6.9)^1^
	16	µmol/mmolTC	0.05 (0.04; 0.06)	0.05 (0.04; 0.06)	0.00 (0.00; 0.00)^3^	−4.8 (−8.7; −0.95)
*Fat-soluble vitamins*						
α-Tocopherol	55	µmol/L	35.76 (33.63; 37.89)	33.16 (31.27; 35.06)	−2.43 (−2.81; −2.05)^1^	−7.1 (−8.0; −6.2)^1^
	54	µmol/mmolTC	5.91 (5.63; 6.18)	5.92 (5.63; 6.20)	−0.01 (−0.06; 0.03)	−0.3 (−1.1; 0.5)
γ-Tocopherol	22	µmol/L	3.00 (2.55; 3.46)	2.91 (2.39; 3.43)	−0.17 (−0.24; −0.11)^1^	−6.9 (−9.8; −3.9)^1^
	21	µmol/mmolTC	0.51 (0.42; 0.60)	0.53 (0.43; 0.64)	0.00 (−0.01; 0.01)	0.2 (−3.2; 3.6)
Retinol	46	µmol/L	2.65 (2.14; 3.16)	2.50 (2.08; 2.91)	−0.02 (−0.04; 0.00)	−0.8 (−1.8; 0.2)
Vitamin D	28	nmol/L	57.56 (51.47; 63.65)	57.95 (51.10; 64.80)	−0.45 (−0.91; 1.81)	1.4 (−1.7; 4.4)
*Lipid and lipoproteins*						
TC	63	mmol/L	5.96 (5.80; 6.12)	5.54 (5.39; 5.69)	−0.39 (−0.42; −0.36)^1^	−6.5 (−7.1; −6.0)^1^
LDL-C	64	mmol/L	3.87 (3.72; 4.01)	3.50 (3.36; 3.64)	−0.35 (−0.38; −0.32)^1^	−9.0 (−9.8; −8.2)^1^
HDL-C	62	mmol/L	1.44 (1.40; 1.48)	1.43 (1.38; 1.48)	0.00 (−0.00; 0.01)	0.2 (−0.4; 0.7)
TAG	60	mmol/L	1.37 (1.30; 1.43)	1.30 (1.23; 1.37)	−0.06 (−0.08; −0.04)^1^	−4.6 (−6.0; −3.2)^1^

Expressed as means (95 % CI)

*HDL-C* high-density lipoprotein cholesterol, *LDL-C* low-density lipoprotein cholesterol, *PS* plant sterols or plant stanols, *TC* total cholesterol, *TAG* triacylglycerol

^1^ *P* < 0.0001; ^2^ *P* < 0.01; ^3^ *P* < 0.05

^a^For parallel studies, the weighted average baseline concentrations were calculated based on the baseline concentrations in the active and placebo groups and for crossover studies, the end-of-intervention concentrations of the placebo periods were used

^b^For parallel studies, the weighted average concentrations after PS intervention were calculated based on the concentrations after PS intervention in the active groups and for crossover studies, the end-of-intervention concentrations of the active periods were used

TC concentrations were significantly (*P* < 0.0001) decreased on average by 0.39 mmol/L (−6.5 %) and LDL-C concentrations by 0.35 mmol/L (−9.0 %). HDL-C concentrations did not change after plant sterol or plant stanol consumption (0.2 %), while TAG concentrations were significantly (*P* < 0.0001) decreased by 0.06 mmol/L (−4.6 %) (Table 1).

**Fig. 2:**
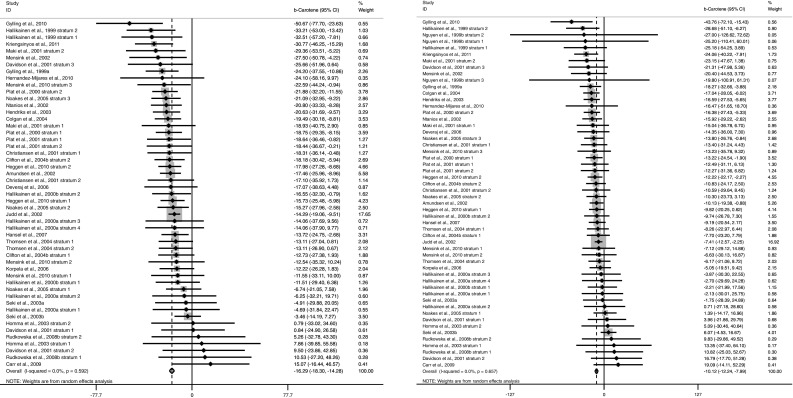
Forest plot relative change (expressed as summary estimates and 95 % CIs) in non-standardized (*left panel*) and TC-standardized (*right panel*) plasma β-carotene concentrations. The *solid squares* represent the weight of individual study arms, and the pooled effect estimate is represented as a *diamond*. Heterogeneity is assessed using Cochran’s *Q* test (*P* < 0.1 indicates significant heterogeneity) and quantified by I^2^, where >50 % indicates substantial heterogeneity

**Fig. 3:**
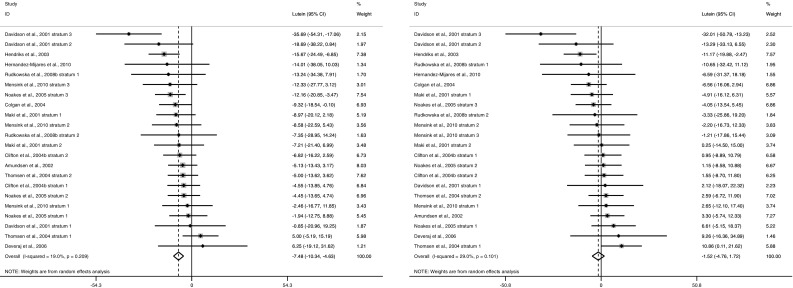
Forest plot relative change (expressed as summary estimates and 95 % CIs) in non-standardized (*left panel*) and TC-standardized (*right panel*) plasma lutein concentrations. The *solid squares* represent the weight of individual study arms, and the pooled effect estimate is represented as a *diamond*. Heterogeneity is assessed using Cochran’s *Q* test (*P* < 0.1 indicates significant heterogeneity) and quantified by I^2^, where >50 % indicates substantial heterogeneity

**Fig. 4:**
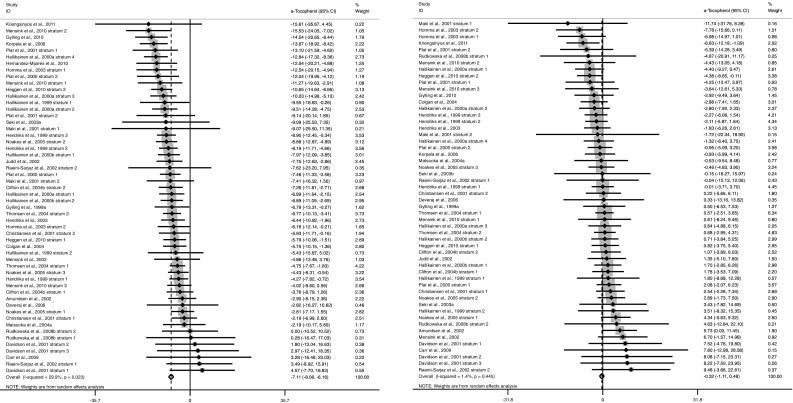
Forest plot relative change (expressed as summary estimates and 95 % CIs) in non-standardized (*left panel*) and TC-standardized (*right panel*) plasma α-tocopherol concentrations. The *solid squares* represent the weight of individual study arms, and the pooled effect estimate is represented as a *diamond*. Heterogeneity is assessed using Cochran’s *Q* test (*P* < 0.1 indicates significant heterogeneity) and quantified by I^2^, where >50 % indicates substantial heterogeneity

Figures 2, 3 and 4 show forest plots of the relative non-standardized and TC-standardized changes for β-carotene, lutein and α-tocopherol. Funnel plots for the relative non-standardized and TC-standardized changes in β-carotene, lutein and α-tocopherol are shown in Online Supplemental Material Figs. 1–3. Visual inspection and calculation of I^2^ statistics (*P* < 0.05) indicated substantial heterogeneity in the relative non-standardized changes of α-tocopherol. Heterogeneity was also present for relative non-standardized changes of zeaxanthin and lycopene and for the relative TC-standardized changes of α-carotene and zeaxanthin (data not shown). Publication bias seemed only present for α-carotene (Egger test: *P* (intercept) < 0.05; studies reporting small decreases in plasma changes seemed lacking, data not shown).

### Covariate analyses

Table 2 provides an overview of the covariate analyses for the TC-standardized changes in β-carotene, lutein, α-tocopherol (β-carotene representing the hydrocarbon carotenoids, lutein the oxygenated carotenoids and α-tocopherol the tocopherols) and for TC concentrations. Overall, baseline concentrations did not have a significant impact on the observed changes after plant sterol or plant stanol consumption. Only some trends were observed implying larger absolute reductions in β-carotene and larger absolute and relative reductions in α-tocopherol with higher baseline values. Furthermore, the plant sterol or plant stanol dose did not significantly affect TC-standardized absolute or relative changes in β-carotene, lutein and α-tocopherol. Only study duration (≤4 vs. >4 weeks) resulted in larger TC-standardized absolute and relative reductions in lutein (0.000 vs. −0.002 µmol/mmol TC and 1.0 vs. −5.6 %, respectively), whereas only a trend for such effect was observed for β-carotene. Consumption of plant stanols seemed to have a stronger effect on lowering relative TC-standardized β-carotene concentrations than plant sterols (−14.2 vs. −8.9 %, respectively), while changes in α-tocopherol concentrations showed a trend toward larger reductions after plant stanol vs. sterol consumption. Changes in TC concentrations were also larger after consumption of plant stanols vs. plant sterols (−8.0 vs. −6.1 %, respectively), which is probably related to a higher dose in the plant stanol compared to the plant sterol studies (3.2 vs. 2.1 g/d). In addition, changes in TC concentrations after plant sterol or plant stanol consumption were significantly affected by baseline concentrations (only absolute TC changes) and by the dose of plant sterol or plant stanol intake.

**Table 2:** Covariate analyses of absolute and relative TC-standardized changes in β-carotene, lutein, α-tocopherol and total cholesterol concentrations after plant sterol or plant stanol consumption

Covariate	Subgroup definition	*n*	Change vs. placebo	95 % CI	*P* value between subgroups^1^	Change vs. placebo	95 % CI	*P* value between subgroups
			*Absolute change in β*-*carotene (*µmol/mmolTC*)*			*Relative change in β*-*carotene (%)*		
Baseline	<0.09 µmol/mmoLTC	27	−0.007	(−0.009; −0.005)	0.059	−9.8	(−12.6; −7.0)	0.747
	>0.09 µmol/mmoLTC	27	−0.011	(−0.015; −0.007)		−10.5	(−13.7; −7.3)	
Dose	0.45 ≤ dose ≤ 1.6	16	−0.008	(−0.012; −0.005)	0.985	−8.5	(−12.3; −4.7)	0.587
	1.6 < dose ≤ 2.0	14	−0.008	(−0.013; −0.004)		−9.9	(−14.4; −5.5)	
	2.0 < dose ≤ 3.0	16	−0.007	(−0.010; −0.005)		−11.2	(−15.1; −7.3)	
	3.0 < dose ≤ 9.0	8	−0.008	(−0.014; −0.003)		−13.8	(−21.2; −6.4)	
Duration	≤4 weeks	28	−0.007	(−0.009; −0.005)	0.093	−8.7	(−11.2; −6.1)	0.050
	>4 weeks	26	−0.010	(−0.014; −0.007)		−13.2	(−17.0; −9.5)	
Sterol vs. stanol	Sterol	31	−0.007	(−0.009; −0.005)	0.206	−8.9	(−11.3; −6.5)	0.039
	Stanol	23	−0.010	(−0.014; −0.006)		−14.2	(−18.6; −9.8)	

			*Absolute change in lutein (*µmol/mmolTC*)*			*Relative change in lutein (%)*		
Baseline	<0.06 µmol/mmoLTC	11	0.001	(−0.003; 0.001)	0.656	−2.7	(−6.8; 1.5)	0.416
	>0.06 µmol/mmoLTC	11	0.000	(−0.003; 0.003)		−0.1	(−4.6; 4.3)	
Dose	0.45 ≤ dose ≤ 1.6	10	0.000	(−0.002; 0.001)	0.134	−1.1	(−4.9; 2.7)	0.171
	1.6 < dose ≤ 2.0	4	0.002	(−0.002; 0.006)		3.0	(−4.5; 10.6)	
	2.0 < dose ≤ 3.0	3	0.001	(−0.004; 0.005)		1.6	(−8.2; 11.4)	
	3.0 < dose ≤ 9.0	5	−0.005	(−0.010; −0.001)		−7.8	(−14.6; −0.9)	
Duration	≤4 weeks	11	0.000	(−0.001; 0.002)	0.048	1.0	(−2.6; 4.5)	0.028
	>4 weeks	11	−0.002	(−0.004; 0.000)		−5.6	(−10.2; −0.9)	
Sterol versus stanol	Sterol	19	−0.001	(−0.002; 0.001)	0.954	−1.6	(−4.9; 1.6)	0.785
	Stanol	3	0.000	(−0.009; 0.009)		−0.2	(−9.8; 9.4)	

			*Absolute change in α*-*tocopherol (*µmol/mmolTC*)*			*Relative change in α*-*tocopherol (%)*		
Baseline	< 5.64 µmol/mmoLTC	27	0.014	(−0.039; 0.067)	0.072	0.3	(−0.7; 1.3)	0.074
	> 5.64 µmol/mmoLTC	27	−0.077	(−0.161; 0.007)		−1.2	(−2.4; 0.1)	
Dose	0.45 ≤ dose ≤ 1.6	17	0.008	(−0.064; 0.080)	0.318	−0.1	(−1.3; 1.2)	0.354
	1.6 < dose ≤ 2.0	14	−0.021	(−0.105; 0.063)		−0.5	(−1.9; 1.0)	
	2.0 < dose ≤ 3.0	14	0.031	(−0.067; 0.129)		0.5	(−1.3; 2.2)	
	3.0 < dose ≤ 9.0	9	−0.106	(−0.225; 0.012)		−2.0	(−4.2; 0.2)	
Duration	≤4 weeks	32	−0.011	(−0.064; 0.042)	0.952	−0.3	(−1.2; 0.6)	0.947
	>4 weeks	22	−0.014	(−0.102; 0.074)		−0.3	(−1.9; 1.4)	
Sterol vs. stanol	Sterol	33	0.017	(−0.037; 0.071)	0.062	0.2	(−0.8; 1.1)	0.070
	Stanol	21	−0.076	(−0.157; 0.005)		−1.4	(−2.7; 0.0)	

			*Absolute change in TC (*mmol/L*)*			*Relative change in TC (%)*		
Baseline	<6.1 mmoL/L	30	−0.337	(−0.382; −0.291)	0.002	−6.3	(−7.0; −5.4)	0.364
	>6.1 mmoL/L	33	−0.435	(−0.479; −0.391)		−6.8	(−7.5; −6.0)	
Dose	0.45 ≤ dose ≤ 1.6	21	−0.308	(−0.357; −0.258)	0.001	−5.2	(−5.8; −4.5)	0.000
	1.6 < dose ≤ 2.0	15	−0.414	(−0.473; −0.353)		−6.9	(−7.8; −6.0)	
	2.0 < dose ≤ 3.0	16	−0.414	(−0.474; −0.353)		−7.6	(−8.5; −6.7)	
	3.0 < dose ≤ 9.0	11	−0.468	(−0.543; −0.394)		−8.2	(−9.4; −7.0)	
Duration	≤4 weeks	37	−0.396	(−0.437; −0.356)	0.548	−6.8	(−7.5; −6.1)	0.162
	>4 weeks	26	−0.374	(−0.435; 0.314)		−6.0	(−6.9; −5.1)	
Sterol versus stanol	Sterol	41	−0.365	(−0.404; 0.326)	0.026	−6.1	(−6.7; −5.5)	0.005
	Stanol	22	−0.448	(−0.509; −0.386)		−8.0	(−9.1; −6.8)	

Expressed as means (95 % CI)

*TC* total cholesterol

^1^ *P* value between subgroups <0.05 indicates a significant difference in pooled effect sizes between subgroups

## Discussion

Although there is general consensus about the serum cholesterol-lowering efficacy of consuming foods enriched with plant sterols and stanols, there is an ongoing discussion around the potential effects of these ingredients on plasma fat-soluble vitamin and carotenoid concentrations. Therefore, we here present the first extensive systematic review and meta-analysis to estimate the effects of plant sterol and plant stanol consumption on plasma fat-soluble vitamin and carotenoid concentrations. We observed a decrease in non-standardized (hydrocarbon and oxygenated) carotenoids and in tocopherol concentrations, but not in vitamin D and retinol concentrations. TC-standardized concentrations remained significantly decreased for all individual hydrocarbon carotenoids, while the oxygenated carotenoid concentrations were differently affected and TC-standardized tocopherol concentrations were not changed after plant sterol and plant stanol consumption.

In an earlier less extensive review from 2003 by Katan et al. [10] including eighteen studies, a reduction in TC-standardized β-carotene was found, while TC-standardized concentrations of α-carotene, lycopene, α-tocopherol and absolute concentrations of vitamin D and retinol were not affected by plant sterol and plant stanol consumption. Our data based on 41 studies available in December 2014 are partly in agreement with these earlier findings, since we observed a significant decrease in all TC-standardized hydrocarbon carotenoid concentrations, while Katan et al. only reported a significant decrease in TC-standardized β-carotene but not in TC-standardized α-carotene and lycopene concentrations.

Regarding the interpretation of changes in absolute fat-soluble vitamin and carotenoid concentrations, it needs to be emphasized that these components are transported by lipoproteins (except retinol and vitamin D) [60, 61]. Therefore, a reduction in non-standardized concentrations might be the result of reduced carrier capacity, as evidenced by lower serum cholesterol concentrations after plant sterol or plant stanol intake. For this reason, fat-soluble vitamin and carotenoid concentrations are generally standardized for various plasma lipid fractions. We chose to standardize for TC (reflecting cholesterol in all lipoproteins), since fat-soluble vitamins and carotenoids are not selectively carried in one lipoprotein fraction. After standardization for TC, α- and γ-tocopherol concentrations were not changed, while changes in oxygenated carotenoids became less significant (zeaxanthin and β-cryptoxanthin) or were no longer present (lutein). Reductions in TC-standardized concentrations for all hydrocarbon carotenoids (β-carotene, α-carotene and lycopene) remained significant although relative changes were smaller compared with the non-standardized changes. This suggests that reductions in non-standardized tocopherol concentrations can primarily be explained by a reduction in the number of lipoprotein particles, while changes in non-standardized carotenoid concentrations can only partly be explained by a decrease in carrier capacity. For the lipophilic hydrocarbon carotenoids, other factors should be considered to explain the observed reductions. Analog to cholesterol, plant sterol and plant stanols, fat-soluble vitamins and carotenoids are lipophilic compounds that require solubilization into mixed micelles for intestinal absorption [60, 61]. Plant sterols and plant stanols interfere with micellar sterol composition and probably also interact with incorporation of fat-soluble vitamins and carotenoids in the micelles. This seems particularly evident for the more lipophilic hydrocarbon carotenoids for which the largest reductions in serum concentrations were found after plant sterol or plant stanol intake. Indeed, Plat and Mensink [19] have shown that changes in hydrocarbon (but not in oxygenated) carotenoid concentrations were significantly related to reductions in markers of cholesterol absorption after plant stanol consumption.

Subgroup analyses showed a stronger reduction in relative TC-standardized β-carotene concentrations and a trend toward larger reductions in TC-standardized α-tocopherol concentrations after plant stanol consumption as compared with plant sterol consumption. Changes in TC concentrations were also larger after consumption of plant stanols compared with plant sterols. Plant stanol studies investigated overall higher doses (3.2 vs. 2.1 g/d with plant sterols), which probably explains the larger TC-lowering efficacy. Indeed, subgroup analyses indicated that TC concentrations decreased dose dependently. Even though a dose–response effect was lacking for relative TC-standardized β-carotene concentrations, largest decreases were seen in the highest dose category (3.0 < dose ≤ 9.0 g/d). As plant stanol studies investigated overall higher doses, the difference between plant sterol and stanol studies on relative TC-standardized β-carotene concentrations might be explained by a difference in average dose. Nevertheless, this is still an unexpected finding and a side-by-side comparison would be needed to evaluate a difference between plant sterols and plant stanols on serum β-carotene concentrations in more detail. Baseline concentrations as well as the duration of the interventions did not have a major impact on plasma fat-soluble vitamin and carotenoid concentrations after plant sterol or plant stanol consumption. In addition, as already mentioned, a clear dose–response effect was lacking for fat-soluble vitamin and carotenoid concentrations, while TC concentrations decreased dose dependently after plant sterol or plant stanol consumption. Such a dose-dependent cholesterol lowering has been shown by Mensink et al. [16], where a linear dose–response relationship was reported for plant stanol intake and cholesterol decrease up to 9 g/d. A dose–response relationship for the reduction in LDL-C at least up to intakes of 3 g/d of plant sterols and stanols was also found in the meta-analysis of Ras et al. [3].

Whether the observed reductions in plasma tocopherols and especially carotenoid concentrations are clinically relevant is not known. In fact, ranges of plasma fat-soluble vitamin and carotenoid concentrations that are defined as normal are very wide [62, 63]. The Food and Nutrition Board of the Health and Medicine Division reported, based on data of more than 20.000 subjects who participated in the NHANES 1988–1994 [64], an average plasma β-carotene concentration of 0.35 μmol/L, and 95 % of the subjects had values between 0.10 and 0.86 μmol/L. We observed that β-carotene was reduced from 0.60 μmol/L (95 % CI 0.54; 0.67) to 0.52 μmol/L (95 % CI 0.46; 0.57) after plant sterol and plant stanol consumption; thus, β-carotene concentrations remained within normal ranges. In addition, they reported [64] average concentrations of α-carotene 0.09 (0.02–0.23) μmol/L, β-cryptoxanthin 0.17 (0.06–0.36) μmol/L and lycopene 0.44 (0.18–0.76) μmol/L. Lutein and zeaxanthin were reported together with an average concentration of 0.37 (0.17–0.68) μmol/L. Our observed carotenoid concentrations remained within these reported ranges after plant sterol and plant stanol consumption. Ford et al. [65] reported a mean α-tocopherol concentration of 27.4 (95 % CI 26.7; 28.1) μmol/L and a mean γ-tocopherol concentration of 4.8 (95 % CI 4.5; 5.1) μmol/L in >4000 subjects who participated in NHANES 1999–2000. We observed overall higher α-tocopherol concentrations and lower γ-tocopherol concentrations, which can be explained by a higher intake of γ-tocopherol in the US diet compared to a higher α-tocopherol intake in the European diet. Nevertheless, our observed total tocopherol concentration (α- and γ-tocopherol) after plant sterol and stanol intake remained within ranges as presented for 450 healthy subjects [63] with a median value of 28.6 (18.4–46.0) μmol/L and >20.000 subjects who participated in NHANES 1988–1994 with a mean concentration of 25.3 (14.6–43.6) μmol/L [64].

For CVD, the overall notion is that a healthy diet with high intakes of fruit and vegetables may lower the risk to develop CVD. Whether this effect is related to antioxidants is not known, also because clinical trials do not support a causal role for antioxidant supplementation in the prevention of chronic diseases such as CVD [8, 9]. In contrast, some prospective cohort studies have reported inverse associations between absolute carotenoid concentrations and the risk to develop CVD or cancers [5, 7, 66], although such relationships have not been observed in all studies [67, 68]. In addition, associations do not imply causality, complicating the interpretation of decreased plasma carotenoid concentrations after plant sterol or stanol consumption. Except for a possible role as antioxidant, it is well established that carotenoids (particularly β-carotene) are a precursor for the synthesis of vitamin A [69]. However, serum vitamin A (retinol) concentrations were not changed after plant sterol or plant stanol intake. A more consistent association is present for dietary lutein and zeaxanthin intake, where higher plasma concentrations are associated with a reduced risk to develop advanced AMD [6]. Although data are limited, active modulation of plasma lutein concentrations by supplementation may also affect AMD and CVD risk [70, 71].

The reported changes in fat-soluble vitamins and carotenoids should also be placed in context of other (dietary) interventions where intestinal absorption of nutrients or carrier capacity in plasma is potentially affected. The addition of dietary fibers such as oat and barley β-glucans to the diet reduces serum cholesterol concentrations [72, 73] but have also been reported to affect fat-soluble vitamin and carotenoid concentrations in humans [74, 75]. Moreover, Kerckhoffs [76] showed a reduction of 7.2 % (*P* < 0.05) in lipid-standardized hydrocarbon carotenoid concentrations after consumption of 5 g/d of oat β-glucan for 2 weeks in 25 healthy subjects. In six healthy women, adding wheat bran to an antioxidant mixture reduced the postprandial response of lycopene and lutein concentrations, while pectin, guar and alginate (water-soluble fibers) reduced β-carotene, lycopene and lutein concentrations [77]. The postprandial response of α-tocopherol was not changed after consumption of these dietary fibers, which probably relates to the lower lipophilicity of α-tocopherol [77]. Besides cholesterol-lowering foods, pharmacological intervention aimed to reduce serum cholesterol concentrations might also have an effect on fat-soluble vitamin and carotenoid concentrations. HMG-CoA reductases inhibitors (statins) are the most frequently prescribed cholesterol-lowering drugs worldwide, and reductions up to 50 % can be achieved with statin therapy [78]. However, despite their effects on serum cholesterol concentrations, concentrations of fat-soluble vitamin and carotenoid are not reduced. If anything, lipoprotein particles become enriched in vitamin/carotenoids after statin therapy [76, 79, 80]. Another cholesterol-lowering drug is ezetimibe, which reduces intestinal uptake of cholesterol by acting on the intestinal cholesterol transporter NPC1L1 [81]. Ezetimbe is a relatively new approach to lower cholesterol concentrations and human data describing whether ezetimibe therapy has an effect on fat-soluble vitamin and carotenoid concentrations is lacking. There are, however, some indications from in vitro studies that ezetimibe affects the intestinal transport of carotenoids and that this effect decreases with increasing polarity of the carotenoids [82]. Other compounds that interfere with cholesterol absorption (i.e., neomycin), fat absorption (olestra) and bile acid reabsorption (cholestyramine) have also been shown to reduce tocopherol and carotenoid concentrations. Reported effects were at least as large or larger than observed for plant sterols or stanols [83–85].

The question is whether the observed changes in plasma fat-soluble vitamins and carotenoids during consumption of plant sterol or stanol ester-enriched foods can be corrected for? It has been indeed reported that reductions in plasma carotenoid concentrations can be prevented by adopting a healthy diet with at least five servings per day of vegetables and/or fruit as recommend by many guidelines for a healthy diet [48, 86]. For instance, the recently released AHA/ACC lifestyle management guidelines for healthy living emphasize the consumption of vegetables, fruits and whole grains for adults who wish to lower their LDL-C concentrations [87]. Other guidelines like the one of the ESC/EAS also mention the consumption of plant sterol- and plant stanol-enriched foods in order to further enhance the cholesterol-lowering effect of a healthy diet [88]. Finally, consumption of a cholesterol-lowering dietary portfolio, which was rich in plant sterols, viscous fibers, soy proteins and nuts for 6 months, reduced LDL-C concentrations (−13.1 %, CI −16.7 to −9.5 %) without affecting TC-standardized tocopherol and carotenoid concentrations [89]. Again, this demonstrates that it is possible to counteract a decrease in TC-standardized carotenoid concentrations after consumption of plant sterol and plant stanol-enriched foods by changing to a recommended dietary pattern.

The current meta-analysis included study evidence based on a variety of experimental study designs regarding aspects such as duration of intervention, food products used and different population groups, which could have contributed to the observed heterogeneity between the studies. However, heterogeneity was only present in a few measured parameters with relative small I^2^ statistics, suggesting little variability between the included studies. In addition, we here report comparable changes in serum cholesterol and lipoprotein concentrations as found in previously published meta-analyses [1–3], implying that the included studies in this meta-analyses are representative of all available studies in the literature that have been performed with plant sterols and plant stanols.

In summary, this meta-analysis including data of 41 RCTs showed that consumption of plant sterols and plant stanols reduces TC-standardized hydrocarbon carotenoid concentrations (β-carotene, α-carotene and lycopene), differently affects TC-standardized oxygenated carotenoid concentrations (reduction in zeaxanthin and β-cryptoxanthin but not in lutein) and does not affect TC-standardized tocopherol concentrations or absolute retinol and vitamin D concentrations. Observed levels in this meta-analysis remained within ranges that are considered to be normal and there are no strong indications that the observed decreases have negative health implications.

## Electronic supplementary material

Below is the link to the electronic supplementary material.

Supplementary material 1 (DOCX 755 kb)
