# Supplementary material for: Plasma fat-soluble vitamin and carotenoid concentrations after plant sterol and plant stanol consumption: a meta-analysis of randomized controlled trials
Source: Eur J Nutr. 2016 Sep 3;56(3):909–23. doi: 10.1007/s00394-016-1289-7 (PMC5346416; doi:10.1007/s00394-016-1289-7)
Supplement: Supplementary file 1 — Supplementary material 1 (DOCX 755 kb) [file 394_2016_1289_MOESM1_ESM.docx]

**Plasma fat-soluble vitamin and carotenoid concentrations after plant sterol and plant stanol consumption – a meta-analysis of randomized controlled trials**

Sabine Baumgartner^1^*, Rouyanne T. Ras^2^*, Elke A. Trautwein^2^, Ronald P. Mensink^1^ and Jogchum Plat^1^

^1^Department of Human Biology, NUTRIM school of Nutrition and Translational Research in Metabolism, Maastricht University, Maastricht, the Netherlands

^2^Unilever R&D Vlaardingen, Vlaardingen, the Netherlands

*S. Baumgartner and R.T. Ras contributed equally

Address correspondence to S. Baumgartner, PO Box 616, 6200 MD, Maastricht, the Netherlands. E-mail: sabine.baumgartner@maastrichtuniversity.nl; Telephone: +31 0 433882113

Supplemental Table 1A-C: Overview of parallel studies

Supplemental Table 2A-C: Overview of crossover studies

Supplemental Figure 1: Funnel plot relative change in non-standardized (left panel) and TC-standardized (right panel) plasma β-carotene concentrations

Supplemental Figure 2: Funnel plot relative change in non-standardized (left panel) and TC-standardized (right panel) plasma lutein concentrations

Supplemental Figure 3: Funnel plot relative change in non-standardized (left panel) and TC-standardized (right panel) plasma α-tocopherol concentrations

Supplemental Table 1A-C: Overview of parallel studies

| **Panel A** | **Subject characteristics** | | | | | **Treatment characteristics** | | | **Total cholesterol** | |
| --- | --- | --- | --- | --- | --- | --- | --- | --- | --- | --- |
| **Reference information** | **Sample size control (n)** | **Sample size active (n)** | **Gender (%male)** | **Age (y)** | **BMI (kg/m2)** | **Sterol or stanol** | **Dose (g/d)^a^** | **Duration (days)** | **Absolute change (mmol/L)** | **Relative change (%)** |
| Carr et al. 2009 | 16 | 16 | 50.0 | 37.1 | 25.5 | sterol | 1.8 | 28 | -0.26 | -3.8 |
| Christiansen et al. 2001 stratum 1 | 46 | 46 | 35.5 | 50.7 | 25.9 | sterol | 1.5 | 182 | -0.34 | -5.1 |
| Christiansen et al. 2001 stratum 2 | 46 | 42 | 35.5 | 50.7 | 26.3 | sterol | 3.0 | 182 | -0.44 | -6.6 |
| Davidson et al. 2001 stratum 1^b^ | 21 | 21 | 52.4 | 45.1 | -^c^ | sterol | 3.0 | 56 | -0.16 | -3.0 |
| Davidson et al. 2001 stratum 2^b^ | 21 | 19 | 50.0 | 47.3 | - | sterol | 6.0 | 56 | -0.34 | -6.3 |
| Davidson et al. 2001 stratum 3^b^ | 21 | 23 | 54.6 | 46.1 | - | sterol | 9.0 | 56 | -0.28 | -5.3 |
| Deveraj et al. 2006 | 36 | 36 | 43.1 | 46.0 | 24.5 | sterol | 2.0 | 56 | -0.16 | -2.8 |
| Gylling et al. 2010 | 24 | 25 | 34.7 | 61.8 | 25.4 | stanol | 8.8 | 70 | -0.69 | -12.0 |
| Hallikainen et al. 1999 stratum 1 | 17 | 18 | 40.0 | 43.3 | 25.6 | stanol | 2.3 | 56 | -0.72 | -10.4 |
| Hallikainen et al. 1999 stratum 2 | 17 | 20 | 32.4 | 44.5 | 24.9 | stanol | 2.2 | 56 | -0.49 | -7.9 |
| Hansel et al. 2007 | 99 | 95 | 67.0 | 48.9 | 23.6 | sterol | 1.6 | 42 | -0.30 | -4.8 |
| Hendriks et al. 2003 | 96 | 89 | 49.0 | 48.0 | 24.9 | sterol | 1.6 | 364 | -0.27 | -4.6 |
| Hernandez-Mijares et al. 2010 | 24 | 31 | 27.3 | 51.8 | 28.2 | sterol | 2.0 | 84 | -0.44 | -7.2 |
| Homma et al. 2003 stratum 1^d^ | 35 | 34 | 36.2 | 46.5 | 23.5 | stanol | 2.0 | 28 | -0.34 | -5.6 |
| Homma et al. 2003 stratum 2^d^ | 35 | 36 | 38.0 | 47.5 | 24.0 | stanol | 3.0 | 28 | -0.29 | -4.9 |
| Korpela et al. 2006^e^ | 82 | 82 | 21.3 | 57.3 | 27.0 | sterol | 1.9 | 42 | -0.44 | -6.9 |
| Kriengsinyos et al. 2011 | 58 | 60 | 32.2 | 39.9 | 24.2 | stanol | 2.0 | 42 | -0.48 | -7.6 |
| Maki et al. 2001 stratum 1^d,f^ | 92 | 92 | 44.0 | 58.1 | 27.5 | sterol | 1.1 | 35 | -0.26 | -4.3 |
| Maki et al. 2001 stratum 2^d,f^ | 92 | 40 | 42.4 | 58.4 | 27.3 | sterol | 2.2 | 35 | -0.47 | -7.5 |
| Mannarino et al. 2009 | 56 | 60 | 44.0 | 50.1 | 25.0 | sterol | 1.6 | 42 | -0.40 | -5.8 |
| Masuda et al. 2007a | 22 | 22 | 34.1 | 46.5 | 22.6 | sterol | 2.4 | 28 | -0.47 | -8.2 |
| Masuda et al. 2007b | 48 | 49 | 59.8 | 46.7 | 23.1 | sterol | 0.8 | 84 | -0.14 | -2.4 |
| Matsuoka et al. 2004 | 23 | 23 | - | 48.0 | 25.8 | sterol | 0.8 | 84 | -0.45 | -7.6 |
| Mensink et al. 2002^f^ | 30 | 30 | 26.7 | 36.0 | 23.3 | stanol | 3.0 | 28 | -0.43 | -8.7 |
| Mensink et al. 2010 stratum 1^f^ | 22 | 24 | 54.3 | 56.3 | 25.1 | stanol | 3.0 | 28 | -0.31 | -4.7 |
| Mensink et al. 2010 stratum 2^f^ | 22 | 22 | 52.3 | 55.7 | 25.2 | stanol | 6.0 | 28 | -0.47 | -7.0 |
| Mensink et al. 2010 stratum 3^f^ | 22 | 25 | 53.2 | 56.1 | 25.0 | stanol | 9.0 | 28 | -0.79 | -12.1 |
| Nguyen et al. 1999 stratum 1^d^ | 80 | 79 | 43.4 | 53.4 | 28.0 | stanol | 3.0 | 56 | -0.3 | -5.0 |
| Nguyen et al. 1999 stratum 2^d^ | 80 | 77 | 47.8 | 54.1 | 27.5 | stanol | 3.0 | 56 | -0.43 | -7.1 |
| Nguyen et al. 1999 stratum 3^d^ | 80 | 82 | 48.8 | 52.9 | 28.3 | stanol | 2.0 | 56 | -0.27 | -4.4 |
| Plat et al 2001 stratum 1^f^ | 42 | 36 | 37.2 | 33.0 | 22.6 | stanol | 3.8 | 56 | -0.39 | -7.8 |
| Plat et al 2001 stratum 2^f^ | 42 | 34 | 36.8 | 33.0 | 23.2 | stanol | 4.0 | 56 | -0.39 | -7.8 |
| Seki et al. 2003a | 28 | 32 | 100.0 | 39.1 | 24.2 | sterol | 0.5 | 84 | -0.16 | -3.0 |
| Seki et al. 2003b | 11 | 11 | 100.0 | 41.2 | 24.2 | sterol | 1.3 | 28 | -0.43 | -9.5 |

| **Panel B** | **Plasma carotenoid/vitamin** | | | | | | | | | | | |
| --- | --- | --- | --- | --- | --- | --- | --- | --- | --- | --- | --- | --- |
|  | **α-Carotene** | | **β-Carotene** | | **Lycopene** | | **Lutein** | | **Zeaxanthin** | | **β-Cryptoxanthin** | |
| **Reference information** | **Absolute change (µmol/L)** | **Relative change (%)** | **Absolute change (µmol/L)** | **Relative change (%)** | **Absolute change (µmol/L)** | **Relative change (%)** | **Absolute change (µmol/L)** | **Relative change (%)** | **Absolute change (nmol/L)** | **Relative change (%)** | **Absolute change (µmol/L)** | **Relative change (%)** |
| Carr et al. 2009 | - | - | 0.05 | 15.1 | - | - | - | - | - | - | - | - |
| Christiansen et al. 2001 stratum 1 | -0.04 | -26.7 | -0.11 | -18.3 | - | - | - | - | - | - | - | - |
| Christiansen et al. 2001 stratum 2 | -0.01 | -3.3 | -0.10 | -17.1 | - | - | - | - | - | - | - | - |
| Davidson et al. 2001 stratum 1^2^ | 0.03 | 19.3 | 0.00 | 0.8 | -0.01 | -2.2 | 0.00 | -0.9 | -24.17 | -20.4 | -0.05 | -25.6 |
| Davidson et al. 2001 stratum 2^2^ | 0.01 | 5.6 | 0.04 | 9.5 | -0.05 | -16.5 | -0.06 | -18.7 | 10.55 | 10.6 | 0.00 | -2.2 |
| Davidson et al. 2001 stratum 3^2^ | -0.01 | -16.3 | -0.17 | -25.7 | -0.04 | -19.0 | -0.10 | -35.7 | 0.00 | -4.5 | -0.01 | -6.7 |
| Deveraj et al. 2006 | 0.01 | 3.5 | -0.10 | -17.1 | -0.12 | -33.9 | 0.01 | 6.3 | - | - | - | - |
| Gylling et al. 2010 | -0.12 | -35.0 | -0.53 | -50.7 | - | - | - | - | - | - | - | - |
| Hallikainen et al. 1999 stratum 1 | 0.05 | 13.9 | -0.50 | -32.5 | -0.18 | -31.7 | - | - | - | - | - | - |
| Hallikainen et al. 1999 stratum 2 | 0.03 | 7.9 | -0.46 | -33.2 | -0.25 | -38.5 | - | - | - | - | - | - |
| Hansel et al. 2007 | - | - | -0.09 | -13.7 | - | - | - | - | - | - | - | - |
| Hendriks et al. 2003 | -0.03 | -40.0 | -0.09 | -20.6 | -0.02 | -7.0 | -0.03 | -15.7 | -9.00 | -16.1 | -0.04 | -18.8 |
| Hernandez-Mijares et al. 2010 | - | - | -0.11 | -24.1 | -0.21 | -23.5 | -0.06 | -14.0 | - | - | -0.04 | 3.5 |
| Homma et al. 2003 stratum 1^4^ | - | - | 0.05 | 7.9 | - | - | - | - | - | - | - | - |
| Homma et al. 2003 stratum 2^4^ | - | - | 0.01 | 0.8 | - | - | - | - | - | - | - | - |
| Korpela et al. 2006^f^ | - | - | -0.08 | -12.2 | - | - | - | - | - | - | - | - |
| Kriengsinyos et al. 2011 | -0.02 | -25.0 | -0.20 | -30.8 | -0.07 | -31.7 | - | - | - | - | -0.28 | -39.8 |
| Maki et al. 2001 stratum 1^d,f^ | -0.02 | -12.5 | -0.10 | -18.9 | -0.03 | -14.5 | -0.02 | -9.0 | -7.54 | -10.9 | -0.02 | -12.7 |
| Maki et al. 2001 stratum 2^d,f^ | -0.04 | -25.5 | -0.16 | -29.4 | -0.04 | -23.2 | -0.02 | -7.2 | -9.36 | -13.6 | -0.04 | -20.4 |
| Mannarino et al. 2009 | - | - | -0.13^g^ | - | - | - | - | - | - | - | - | - |
| Masuda et al. 2007a | - | - | - | - | - | - | - | - | - | - | - | - |
| Masuda et al. 2007b | - | - | - | - | - | - | - | - | - | - | - | - |
| Matsuoka et al. 2004 | - | - | - | - | - | - | - | - | - | - | - | - |
| Mensink et al. 2002^f^ | -0.01 | -20.0 | -0.11 | -27.5 | -0.09 | -16.9 | - | - | - | - | -0.05 | -16.5 |
| Mensink et al. 2010 stratum 1^f^ | - | - | -0.06 | -11.6 | - | - | -0.01 | -2.5 | -6.85 | -10.5 | - | - |
| Mensink et al. 2010 stratum 2^f^ | - | - | -0.05 | -12.5 | - | - | -0.06 | -8.6 | -14.94 | -18.7 | - | - |
| Mensink et al. 2010 stratum 3^6^ | - | - | -0.10 | -22.6 | - | - | -0.07 | -12.3 | -11.25 | -21.4 | - | - |
| Nguyen et al. 1999 stratum 1^d^ | - | - | - | - | - | - | - | - | - | - | - | - |
| Nguyen et al. 1999 stratum 2^d^ | - | - | - | - | - | - | - | - | - | - | - | - |
| Nguyen et al. 1999 stratum 3^d^ | - | - | - | - | - | - | - | - | - | - | - | - |
| Plat et al 2001 stratum 1^f^ | -0.01 | -17.2 | -0.06 | -18.6 | -0.07 | -13.0 | - | - | - | - | -0.03 | -10.6 |
| Plat et al 2001 stratum 2^f^ | -0.01 | -20.8 | -0.06 | -18.4 | -0.15 | -26.7 | - | - | - | - | -0.05 | -12.3 |
| Seki et al. 2003a | - | - | -0.03 | -4.9 | - | - | - | - | - | - | - | - |
| Seki et al. 2003b | - | - | -0.02 | -3.5 | - | - | - | - | - | - | - | - |

| **Panel C** | **Plasma carotenoid/vitamin** | | | | | | | |
| --- | --- | --- | --- | --- | --- | --- | --- | --- |
|  | **α-Tocopherol** | | **γ-Tocopherol** | | **Retinol** | | **Vitamin D** | |
| **Reference information** | **Absolute change (µmol/L)** | **Relative change (%)** | **Absolute change (µmol/L)** | **Relative change (%)** | **Absolute change (µmol/L)** | **Relative change (%)** | **Absolute change (nmol/L)** | **Relative change (%)** |
| Carr et al. 2009 | 1.10 | 3.3 | -5.30 | -38.4 | 0.51 | 17.1 | - | - |
| Christiansen et al. 2001 stratum 1 | -0.60 | -2.2 | - | - | -0.03 | -1.6 | - | - |
| Christiansen et al. 2001 stratum 2 | -2.00 | -5.9 | - | - | -0.03 | -1.5 | - | - |
| Davidson et al. 2001 stratum 1^b^ | 1.29 | 4.6 | -0.26 | -6.1 | -0.28 | -15.5 | 6.56 | 9.6 |
| Davidson et al. 2001 stratum 2^b^ | 0.53 | 1.8 | -0.36 | -9.2 | -0.20 | -11.3 | 7.81 | 13.0 |
| Davidson et al. 2001 stratum 3^b^ | 0.63 | 3.0 | -0.61 | -12.3 | -0.17 | -10.1 | 8.75 | 12.3 |
| Deveraj et al. 2006 | -1.00 | -2.8 | - | - | - | - | - | - |
| Gylling et al. 2010 | -6.01 | -14.5 | -0.29 | -15.6 | 0.01 | 0.6 | 0.97 | 0.3 |
| Hallikainen et al. 1999 stratum 1 | -5.09 | -9.5 | - | - | -0.05 | -1.7 | 2.36 | 15.1 |
| Hallikainen et al. 1999 stratum 2 | -2.46 | -5.4 | - | - | 0.00 | -0.2 | -7.05 | -9.8 |
| Hansel et al. 2007 | - | - | - | - | - | - | - | - |
| Hendriks et al. 2003 | -1.86 | -6.4 | - | - | 0.03 | 1.6 | -6.00 | -7.4 |
| Hernandez-Mijares et al. 2010 | - | - | - | - | - | - | - | - |
| Homma et al. 2003 stratum 1^d^ | -4.80 | -12.5 | - | - | 0.70 | 6.1 | - | - |
| Homma et al. 2003 stratum 2^d^ | -4.80 | -12.5 | - | - | 0.80 | 6.3 | - | - |
| Korpela et al. 2006^e^ | -2.55 | -7.7 | -0.03 | -1.5 | -0.05 | -3.0 | 1.25 | 3.1 |
| Kriengsinyos et al. 2011 | -3.68 | -13.7 | -0.15 | -2.3 | 0.05 | 2.9 | - | - |
| Maki et al. 2001 stratum 1^d,f^ | -6.88 | -15.6 | 0.17 | 4.6 | -0.19 | -8.2 | -5.81 | -6.6 |
| Maki et al. 2001 stratum 2^d,f^ | -3.79 | -9.1 | -0.02 | 0.9 | 0.00 | 0.2 | -0.20 | -1.4 |
| Mannarino et al. 2009 | - | - | - | - | - | - | - | - |
| Masuda et al. 2007a | - | - | - | - | -0.09 | -4.0 | -0.80 | -3.5 |
| Masuda et al. 2007b | - | - | - | - | 0.04 | 1.8 | -1.59 | -4.6 |
| Matsuoka et al. 2004 | -2.55 | -7.4 | - | - | 0.02 | 1.1 | 4.75 | 8.7 |
| Mensink et al. 2002^f^ | -0.58 | -2.2 | - | - | - | - | - | - |
| Mensink et al. 2010 stratum 1^f^ | -1.86 | -4.9 | - | - | - | - | - | - |
| Mensink et al. 2010 stratum 2^f^ | -4.64 | -11.3 | - | - | - | - | - | - |
| Mensink et al. 2010 stratum 3^f^ | -5.80 | -15.5 | - | - | - | - | - | - |
| Nguyen et al. 1999 stratum 1^d^ | - | - | - | - | 0.10^g^ | - | 1.00^g^ | - |
| Nguyen et al. 1999 stratum 2^d^ | - | - | - | - | 0.04^g^ | - | -2.25^g^ | - |
| Nguyen et al. 1999 stratum 3^d^ | - | - | - | - | 0.02^g^ | - | -0.75^g^ | - |
| Plat et al 2001 stratum 1^f^ | -2.87 | -12.0 | - | - | -0.03 | -1.4 | - | - |
| Plat et al 2001 stratum 2^f^ | -3.20 | -13.1 | - | - | -0.04 | -2.0 | - | - |
| Seki et al. 2003a | 0.00 | 0.0 | - | - | -0.07 | -2.7 | - | - |
| Seki et al. 2003b | -2.32 | -9.1 | - | - | 0.11 | 5.4 | - | - |

Footnotes

^a^Plant sterol or plant stanol dose expressed as free equivalents. Carr et al. 2009 reported plant sterol dose as esters; the amount of free equivalents was calculated assuming that 40% of plant sterol esters are fatty acids and 60% are plant sterols

^b^Average concentrations of week 4 and 8 reported for α-carotene, β-carotene, lycopene, lutein, zeaxanthin and β-cryptoxanthin. Concentrations of week 8 are reported for α-tocopherol, γ-tocopherol, retinol and vitamin D.

^c^No data reported

^d^The following papers reported plasma carotenoid/vitamin concentrations in a subset of the total number of subjects included in the study: Homma et al. 2003 (n=35 for β-carotene and retinol), Maki et al. 2001 (n=71 for retinol, α-tocopherol, γ-tocopherol and vitamin D, n=190 for α-carotene, β-carotene, lycopene, lutein, zeaxanthin, β-cryptoxanthin) and Nguyen et al. 1999b (n=73 for β-carotene and retinol and n=290 for vitamin D).

^e^The dose of plant sterols was calculated by taking the average of 2, 2, and 1.65 g/d which were the doses used in the different foods

^f^Non-standardized plasma carotenoid/vitamin data were obtained from the original authors: Maki et al 2001, Mensink et al. 2002, Mensink et al. 2010 and Plat et al. 2001

^g^Extracted from paper since baseline and/or endpoint values are not reported

Supplemental Table 2A-C: Overview of crossover studies

| **Panel A** | **Subject characteristics** | | | | **Treatment characteristics** | | | **Total cholesterol** | |
| --- | --- | --- | --- | --- | --- | --- | --- | --- | --- |
| **Reference information** | **Sample size (n)** | **Gender (%male)** | **Age (y)** | **BMI (kg/m2)** | **Sterol or stanol** | **Dose (g/d)^a^** | **Duration (days)** | **Absolute change (mmol/L)** | **Relative change (%)** |
| Amundsen et al. 2002 | 38 | 50.0 | 10.5 | 19.0 | sterol | 1.60 | 56 | -0.61 | -8.2 |
| Chen et al. 2009 | 22 | 59.1 | 51.7 | 28.0 | sterol | 3.30 | 28 | -0.49 | -9.0 |
| Clifton et al. 2004 stratum 1^b^ | 58 | 39.7 | 54.0 | 26.2 | sterol | 1.60 | 21 | -0.35 | -5.4 |
| Clifton et al. 2004 stratum 2^b^ | 58 | 39.7 | 54.0 | 26.2 | sterol | 1.60 | 21 | -0.53 | -8.2 |
| Colgan et al. 2004 | 48 | 56.3 | 46.0 | 26.3 | sterol | 1.30 | 21 | -0.18 | -3.0 |
| Gylling et al. 1999 | 21 | 0.0 | 52.7 | 25.6 | stanol | 2.40 | 35 | -0.46 | -7.3 |
| Hallikainen et al. 2000a stratum 1 | 22 | 36.4 | 50.5 | 26.3 | stanol | 0.81 | 28 | -0.17 | -2.6 |
| Hallikainen et al. 2000a stratum 2 | 22 | 36.4 | 50.5 | 26.3 | stanol | 1.56 | 28 | -0.45 | -6.9 |
| Hallikainen et al. 2000a stratum 3 | 22 | 36.4 | 50.5 | 26.3 | stanol | 2.29 | 28 | -0.69 | -10.6 |
| Hallikainen et al. 2000a stratum 4 | 22 | 36.4 | 50.5 | 26.3 | stanol | 3.03 | 28 | -0.76 | -11.7 |
| Hallikainen et al. 2000b stratum 1^c^ | 34 | 47.6 | 48.8 | 24.9 | stanol | 2.00 | 28 | -0.58 | -9.5 |
| Hallikainen et al. 2000b stratum 2^c^ | 34 | 47.6 | 48.8 | 24.9 | sterol | 2.00 | 28 | -0.46 | -7.5 |
| Heggen et al. 2010 stratum 1 | 59 | 72.9 | 52.0 | 24.8 | sterol | 2.00 | 28 | -0.40 | -6.6 |
| Heggen et al. 2010 stratum 2 | 59 | 72.9 | 52.0 | 24.8 | sterol | 2.00 | 28 | -0.40 | -6.6 |
| Hendriks et al. 1999 stratum 1^d,e^ | 100 | 42.0 | 37.0 | 22.8 | sterol | 0.83 | 24 | -0.22 | -4.3 |
| Hendriks et al. 1999 stratum 2^d,e^ | 100 | 42.0 | 37.0 | 22.8 | sterol | 1.61 | 24 | -0.32 | -6.2 |
| Hendriks et al. 1999 stratum 3^d,e^ | 100 | 42.0 | 37.0 | 22.8 | sterol | 3.24 | 24 | -0.35 | -6.8 |
| Judd et al. 2002 | 53 | 49.1 | 47.1 | 26.3 | sterol | 2.20 | 21 | -0.39 | -7.4 |
| Noakes et al. 2005 stratum 1^f^ | 39 | 53.9 | 51.5 | 25.9 | sterol | 2.00 | 21 | -0.55 | -8.0 |
| Noakes et al. 2005 stratum 2^f^ | 39 | 53.9 | 51.5 | 25.9 | sterol | 2.00 | 21 | -0.38 | -5.5 |
| Noakes et al. 2005 stratum 3^f^ | 39 | 53.9 | 51.5 | 25.9 | sterol | 4.00 | 21 | -0.58 | -8.5 |
| Ntianos et al. 2002 | 53 | 49.1 | 45.1 | 23.7 | sterol | 1.80 | 21 | -0.32 | -5.8 |
| Plat et al. 2000 stratum 1 | 39 | 28.2 | 31.0 | 22.7 | stanol | 2.50 | 28 | -0.32 | -6.4 |
| Plat et al. 2000 stratum 2 | 39 | 28.2 | 31.0 | 22.7 | stanol | 2.50 | 28 | -0.33 | -6.6 |
| Raeini-Sarjaz et al. 2002 stratum 1 | 15 | 100.0 | -^g^ | - | sterol | 1.92 | 21 | -0.55 | -9.1 |
| Raeini-Sarjaz et al. 2002 stratum 2 | 15 | 100.0 | - | - | stanol | 1.76 | 21 | -0.33 | -5.5 |
| Rudkowska et al. 2008 stratum 1 | 26 | - | 59.6 | 26.4 | sterol | 1.60 | 30 | -0.16 | -2.9 |
| Rudkowska et al. 2008 stratum 2 | 26 | - | 59.6 | 26.4 | sterol | 1.60 | 30 | -0.23 | -4.2 |
| Sierksma et al. 1999 | 76 | 51.3 | 44.0 | 24.4 | sterol | 0.80 | 21 | -0.19 | -3.8 |
| Thomsen et al. 2004 stratum 1 | 69 | 26.1 | 60.0 | 25.9 | sterol | 1.20 | 28 | -0.35 | -5.3 |
| Thomsen et al. 2004 stratum 2 | 69 | 26.1 | 60.0 | 25.9 | sterol | 1.60 | 28 | -0.49 | -7.4 |
| Weststrate et al. 1998 stratum 1^d^ | 95 | 50.0 | 45.0 | 24.2 | sterol | 3.20 | 21 | -0.43 | -8.3 |
| Weststrate et al. 1998 stratum 2^d^ | 95 | 50.0 | 45.0 | 24.2 | stanol | 2.70 | 21 | -0.37 | -7.1 |

| **Panel B** | **Plasma carotenoid/vitamin** | | | | | | | | | | | |
| --- | --- | --- | --- | --- | --- | --- | --- | --- | --- | --- | --- | --- |
|  | **α-Carotene** | | **β-Carotene** | | **Lycopene** | | **Lutein** | | **Zeaxanthin** | | **β-Cryptoxanthin** | |
| **Reference information** | **Absolute change (µmol/L)** | **Relative change (%)** | **Absolute change (µmol/L)** | **Relative change (%)** | **Absolute change (µmol/L)** | **Relative change (%)** | **Absolute change (µmol/L)** | **Relative change (%)** | **Absolute change (nmol/L)** | **Relative change (%)** | **Absolute change (µmol/L)** | **Relative change (%)** |
| Amundsen et al. 2002 | -0.02 | -23.9 | -0.11 | -17.5 | -0.12 | -12.8 | -0.02 | -5.1 | - | - | - | - |
| Chen et al. 2009 | -0.03 | -19.7 | -0.15 | -27.7 | -0.12 | -31.1 | -0.04 | -11.8 | -20.00 | -15.4 | -0.05 | -16.5 |
| Clifton et al. 2004 stratum 1^b^ | 0.00 | 0.0 | -0.07 | -12.7 | -0.06 | -9.0 | -0.02 | -4.5 | - | - | - | - |
| Clifton et al. 2004 stratum 2^b^ | -0.02 | -15.4 | -0.10 | -18.2 | -0.05 | -7.5 | -0.03 | -6.8 | - | - | - | - |
| Colgan et al. 2004 | -0.06 | -24.4 | -0.14 | -19.5 | -0.07 | -10.9 | -0.02 | -9.3 | -7.78 | -10.0 | -0.02 | -6.6 |
| Gylling et al. 1999 | -0.03 | -8.6 | -0.38 | -24.2 | - | - | - | - | - | - | - | - |
| Hallikainen et al. 2000a stratum 1 | 0.00 | 0.0 | -0.03 | -4.7 | 0.20^h^ | 17.9 | - | - | - | - | - | - |
| Hallikainen et al. 2000a stratum 2 | 0.02 | 7.1 | -0.04 | -6.3 | 0.11 | 10.3 | - | - | - | - | - | - |
| Hallikainen et al. 2000a stratum 3 | 0.01 | 3.6 | -0.09 | -14.1 | -0.06 | -5.2 | - | - | - | - | - | - |
| Hallikainen et al. 2000a stratum 4 | 0.01 | 3.6 | -0.09 | -14.1 | 0.13 | 12.1 | - | - | - | - | - | - |
| Hallikainen et al. 2000b stratum 1^c^ | -0.02 | -3.0 | -0.16 | -11.5 | -0.02 | -2.7 | - | - | - | - | - | - |
| Hallikainen et al. 2000b stratum 2^c^ | -0.05 | -7.6 | -0.23 | -16.5 | -0.04 | -5.5 | - | - | - | - | - | - |
| Heggen et al. 2010 stratum 1 | -0.02 | -11.1 | -0.14 | -15.7 | -0.06 | -6.3 | - | - | - | - | - | - |
| Heggen et al. 2010 stratum 2 | -0.03 | -16.7 | -0.16 | -18.0 | -0.11 | -11.6 | - | - | - | - | - | - |
| Hendriks et al. 1999 stratum 1^d,e^ | - | - | - | - | -0.02 | -5.0 | - | - | - | - | - | - |
| Hendriks et al. 1999 stratum 2^d,e^ | - | - | - | - | -0.04 | -10.0 | - | - | - | - | - | - |
| Hendriks et al. 1999 stratum 3^d,e^ | - | - | - | - | -0.03 | -7.5 | - | - | - | - | - | - |
| Judd et al. 2002 | -0.04 | -18.2 | -0.06 | -14.3 | - | - | - | - | - | - | - | - |
| Noakes et al. 2005 stratum 1^f^ | -0.01 | -11.0 | -0.03 | -6.7 | -0.08 | -9.8 | -0.01 | -1.9 | - | - | - | - |
| Noakes et al. 2005 stratum 2^f^ | -0.02 | -16.9 | -0.07 | -15.3 | -0.04 | -5.3 | -0.02 | -4.5 | - | - | - | - |
| Noakes et al. 2005 stratum 3^f^ | -0.02 | -17.7 | -0.09 | -21.1 | -0.12 | -15.6 | -0.05 | -12.2 | - | - | - | - |
| Ntianos et al. 2002 | - | - | -0.12 | -20.8 | - | - | - | - | - | - | - | - |
| Plat et al. 2000 stratum 1 | -0.01 | -20.0 | -0.06 | -18.8 | -0.08 | -11.1 | - | - | - | - | -0.02 | -6.1 |
| Plat et al. 2000 stratum 2 | -0.01 | -20.0 | -0.07 | -21.9 | -0.12 | -16.7 | - | - | - | - | -0.03 | -9.1 |
| Raeini-Sarjaz et al. 2002 stratum 1 | - | - | - | - | - | - | - | - | - | - | - | - |
| Raeini-Sarjaz et al. 2002 stratum 2 | - | - | - | - | - | - | - | - | - | - | - | - |
| Rudkowska et al. 2008 stratum 1 | - | - | 0.04 | 10.5 | -0.14 | -15.6 | -0.09 | -13.2 | - | - | - | - |
| Rudkowska et al. 2008 stratum 2 | - | - | 0.02 | 5.3 | -0.07 | -7.8 | -0.05 | -7.4 | - | - | - | - |
| Sierksma et al. 1999 | - | - | - | - | -0.03 | -13.1 | - | - | - | - | - | - |
| Thomsen et al. 2004 stratum 1 | 0.05 | 17.2 | -0.08 | -13.1 | -0.01 | -2.6 | 0.01 | 5.0 | 0.00 | 0.0 | 0.00 | 0.0 |
| Thomsen et al. 2004 stratum 2 | -0.06 | -20.7 | -0.08 | -13.1 | -0.04 | -10.5 | -0.01 | -5.0 | -10.00 | -33.3 | -0.01 | -5.0 |
| Weststrate et al. 1998 stratum 1^d^ | - | - | - | - | -0.03 | -20.0 | - | - | - | - | - | - |
| Weststrate et al. 1998 stratum 2^d^ | - | - | - | - | -0.04 | -22.2 | - | - | - | - | - | - |

| **Panel C** | **Plasma carotenoid/vitamin** | | | | | | | |
| --- | --- | --- | --- | --- | --- | --- | --- | --- |
|  | **α-Tocopherol** | | **γ-Tocopherol** | | **Retinol** | | **Vitamin D** | |
| **Reference information** | **Absolute change (µmol/L)** | **Relative change (%)** | **Absolute change (µmol/L)** | **Relative change (%)** | **Absolute change (µmol/L)** | **Relative change (%)** | **Absolute change (nmol/L)** | **Relative change (%)** |
| Amundsen et al. 2002 | -1.02 | -2.9 | - | - | 0.00 | 0.0 | - | - |
| Chen et al. 2009 | -4.76 | -12.4 | -0.51 | -8.0 | -0.04 | -1.8 | - | - |
| Clifton et al. 2004 stratum 1^b^ | -1.40 | -3.8 | - | - | -0.03 | -1.3 | - | - |
| Clifton et al. 2004 stratum 2^b^ | -2.70 | -7.3 | - | - | -0.03 | -1.3 | - | - |
| Colgan et al. 2004 | -2.11 | -5.8 | -0.22 | -14.9 | -0.13 | -5.5 | - | - |
| Gylling et al. 1999 | -2.90 | -6.8 | - | - | 0.10 | 4.5 | 0.10 | 0.2 |
| Hallikainen et al. 2000a stratum 1 | -3.21 | -6.9 | -0.08 | -3.8 | 0.03 | 1.0 | 16.01 | 24.8 |
| Hallikainen et al. 2000a stratum 2 | -4.43 | -9.5 | -0.19 | -9.0 | -0.08 | -2.7 | -1.00 | -1.5 |
| Hallikainen et al. 2000a stratum 3 | -4.67 | -10.0 | -0.33 | -15.6 | -0.01 | -0.3 | 2.31 | 3.6 |
| Hallikainen et al. 2000a stratum 4 | -5.98 | -12.8 | -0.27 | -12.7 | -0.03 | -1.0 | 7.31 | 11.3 |
| Hallikainen et al. 2000b stratum 1^c^ | -3.47 | -8.0 | -0.02 | -0.7 | -0.09 | -3.2 | -0.95 | -1.9 |
| Hallikainen et al. 2000b stratum 2^c^ | -3.00 | -6.9 | -0.13 | -4.3 | -0.10 | -3.6 | 1.93 | 3.8 |
| Heggen et al. 2010 stratum 1 | -2.50 | -5.8 | -0.04 | -1.8 | - | - | - | - |
| Heggen et al. 2010 stratum 2 | -4.60 | -10.6 | -0.26 | -11.5 | - | - | - | - |
| Hendriks et al. 1999 stratum 1^d,e^ | -1.20 | -4.3 | - | - | - | - | 3.00 | 4.4 |
| Hendriks et al. 1999 stratum 2^d,e^ | -2.30 | -8.2 | - | - | - | - | 5.00 | 7.4 |
| Hendriks et al. 1999 stratum 3^4,5^ | -2.50 | -8.9 | - | - | - | - | 2.00 | 2.9 |
| Judd et al. 2002 | -1.95 | -6.2 | -0.02 | -0.5 | -0.07 | -4.9 | - | - |
| Noakes et al. 2005 stratum 1^f^ | -1.34 | -4.0 | - | - | 0.00 | 0.1 | - | - |
| Noakes et al. 2005 stratum 2^f^ | -0.94 | -2.8 | - | - | 0.08 | 3.7 | - | - |
| Noakes et al. 2005 stratum 3^f^ | -2.96 | -8.9 | - | - | 0.01 | 0.6 | - | - |
| Ntianos et al. 2002 | - | - | - | - | - | - | - | - |
| Plat et al. 2000 stratum 1 | -1.08 | -4.4 | - | - | -0.02 | -0.9 | - | - |
| Plat et al. 2000 stratum 2 | -1.82 | -7.5 | - | - | 0.02 | 0.9 | - | - |
| Raeini-Sarjaz et al. 2002 stratum 1 | -2.93 | -9.1 | -0.71 | -19.8 | -0.09 | -3.1 | -4.67 | -13.2 |
| Raeini-Sarjaz et al. 2002 stratum 2 | 1.12 | 3.5 | -0.43 | -12.0 | 0.23 | 8.0 | -2.41 | -6.8 |
| Rudkowska et al. 2008 stratum 1 | -4.91 | -7.6 | - | - | - | - | - | - |
| Rudkowska et al. 2008 stratum 2 | 0.18 | 0.3 | - | - | - | - | - | - |
| Sierksma et al. 1999 | - | - | - | - | - | - | - | - |
| Thomsen et al. 2004 stratum 1 | -1.76 | -4.7 | - | - | - | - | - | - |
| Thomsen et al. 2004 stratum 2 | -2.51 | -6.8 | - | - | - | - | - | - |
| Weststrate et al. 1998 stratum 1^d^ | - | - | - | - | - | - | - | - |
| Weststrate et al. 1998 stratum 2^d^ | - | - | - | - | - | - | - | - |

Footnotes

^a^Plant sterol or plant stanol dose expressed as free equivalents. Carr et al. 2009 reported plant sterol dose as esters; the amount of free equivalents was calculated assuming that 40% of plant sterol esters are fatty acids and 60% are plant sterols

^b^Incomplete cross-over design: not all subjects received all treatments (n_control_=58, n_bread_=36, n_milk_=40)

^c^Gender distribution based on 42 subjects (8 drop-outs)

^d^Baseline characteristics based on 100 subjects, balanced incomplete Latin square design (n=80 per group)

^e^Plasma carotenoid/vitamin concentrations reported in a subset of the total number of subjects included in the study (n=60)

^f^Non-standardized plasma carotenoid/vitamin data were obtained from the original authors

^g^No data reported

^h^Lycopene calculated based on separate data for men and women


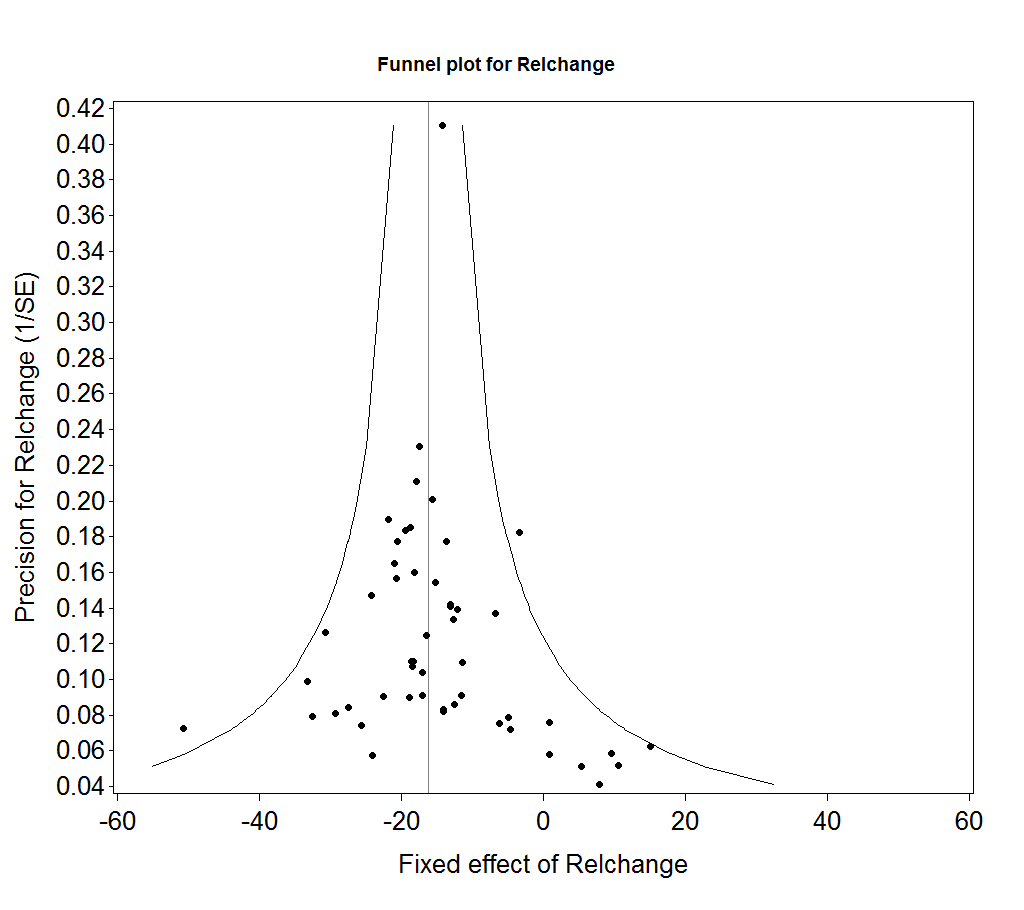

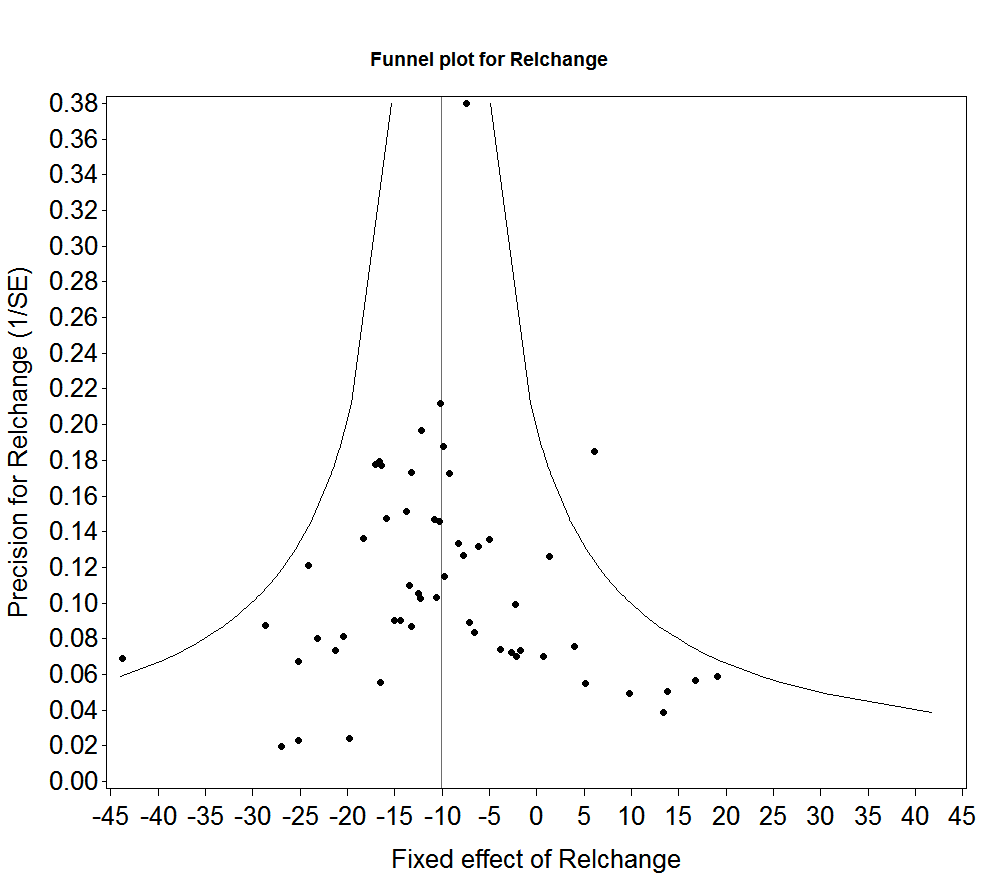


I^2^ = 0.0; P-value = 0.592

Egger test: P-value (intercept) = 0.550

I^2^ = 0.0; P-value = 0.657

Egger test: P-value (intercept) = 0.927

Precision (1/SE)

Supplemental Figure 1: Funnel plot relative change in non-standardized (left panel) and TC-standardized (right panel) plasma β-carotene concentrations


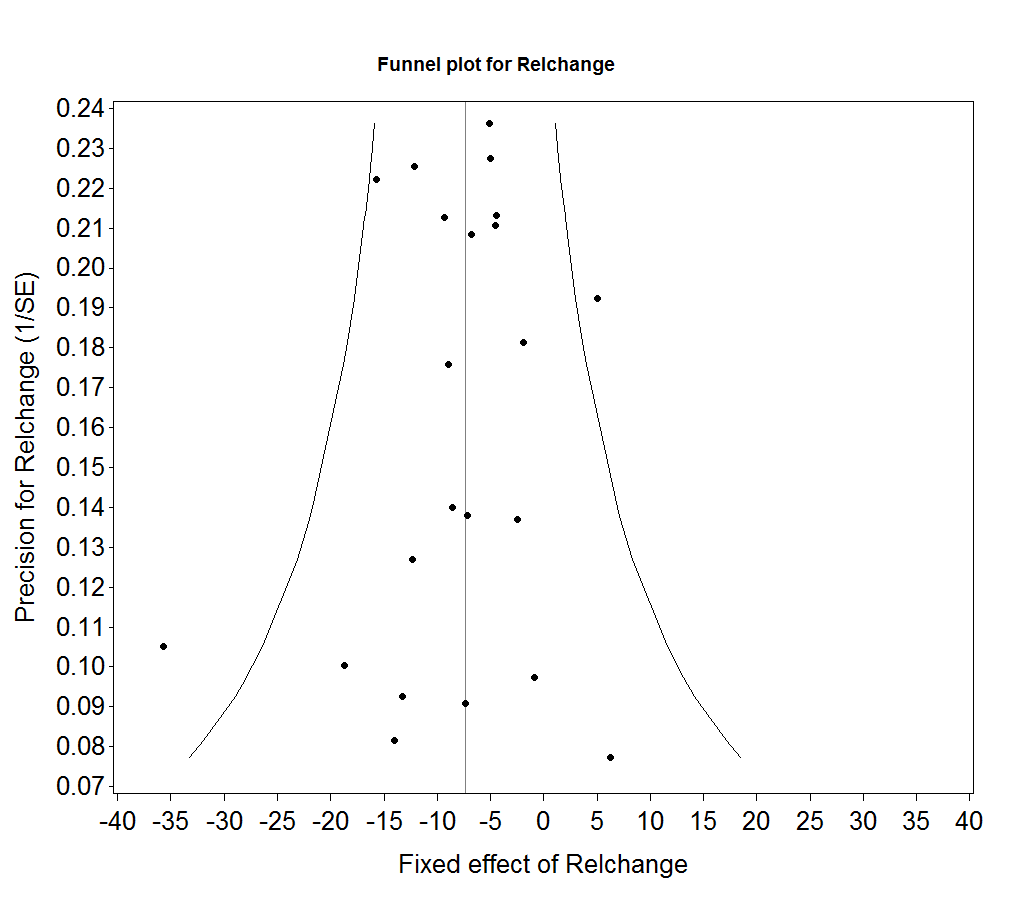

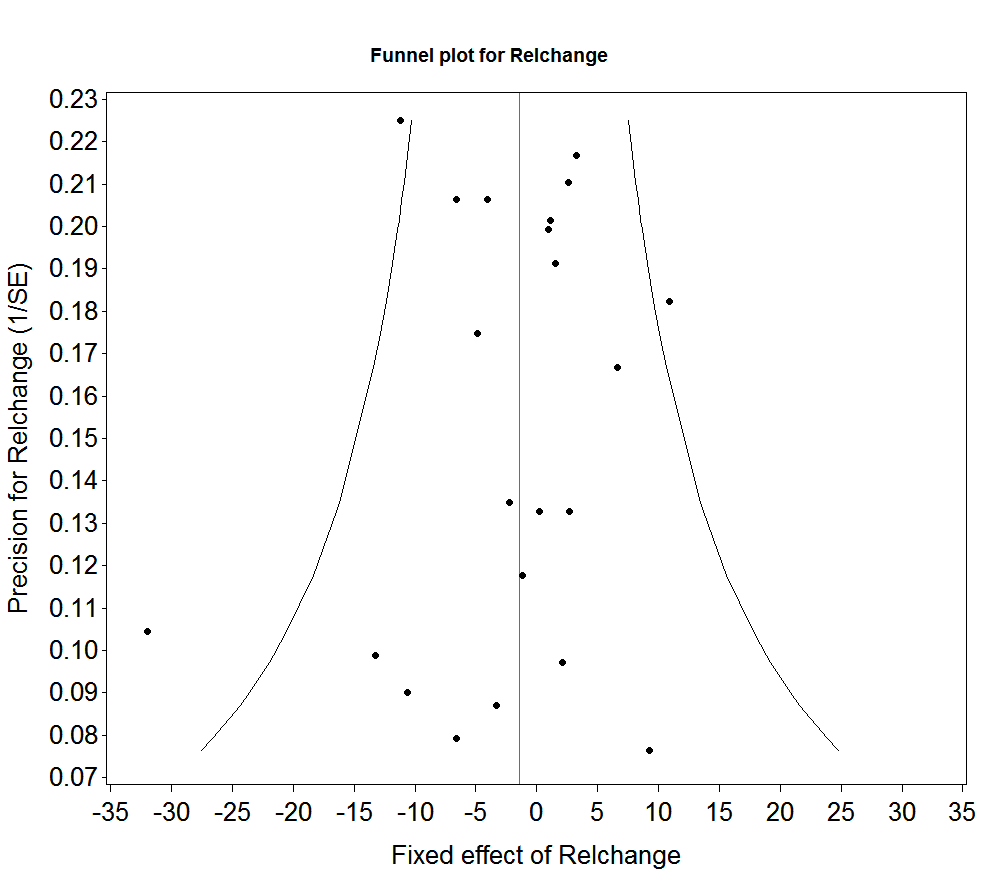


I^2^ = 19.0; P-value = 0.209

Egger test: P-value (intercept) = 0.423

I^2^ = 29.0; P-value = 0.101

Egger test: P-value (intercept) = 0.420

Precision (1/SE)

Supplemental Figure 2: Funnel plot relative change in non-standardized (left panel) and TC-standardized (right panel) plasma lutein concentrations


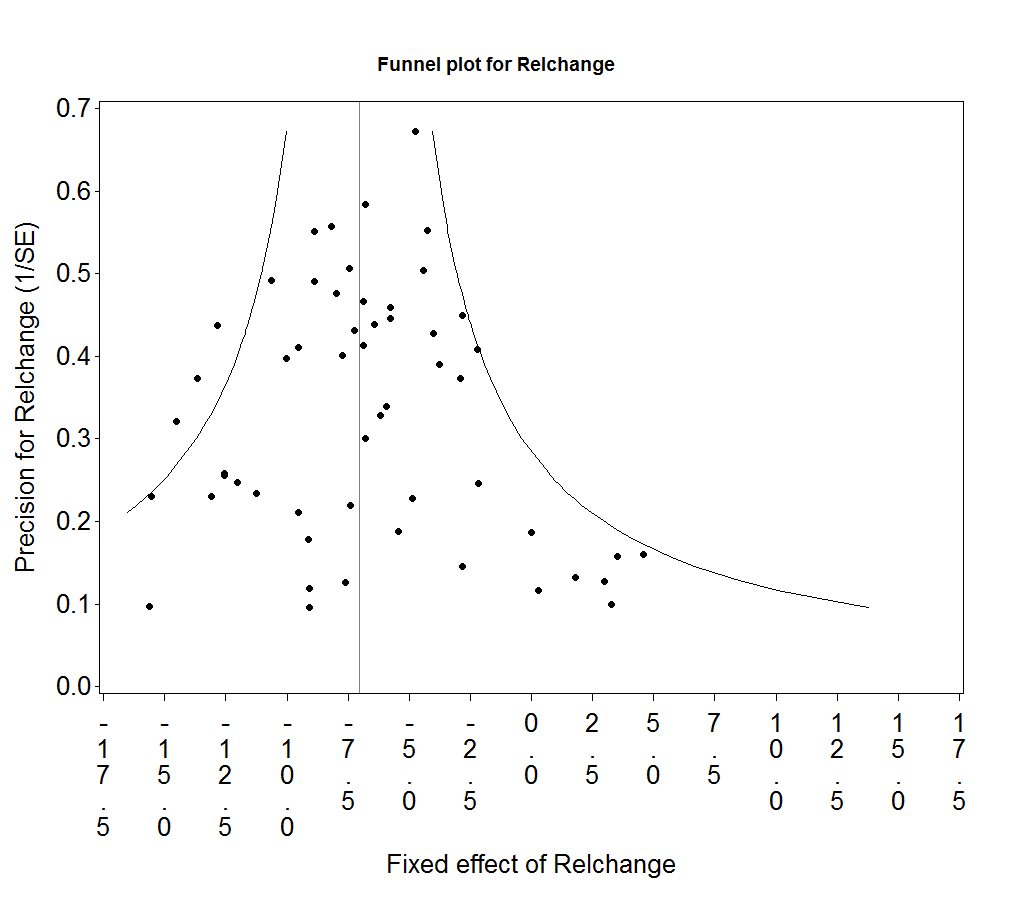

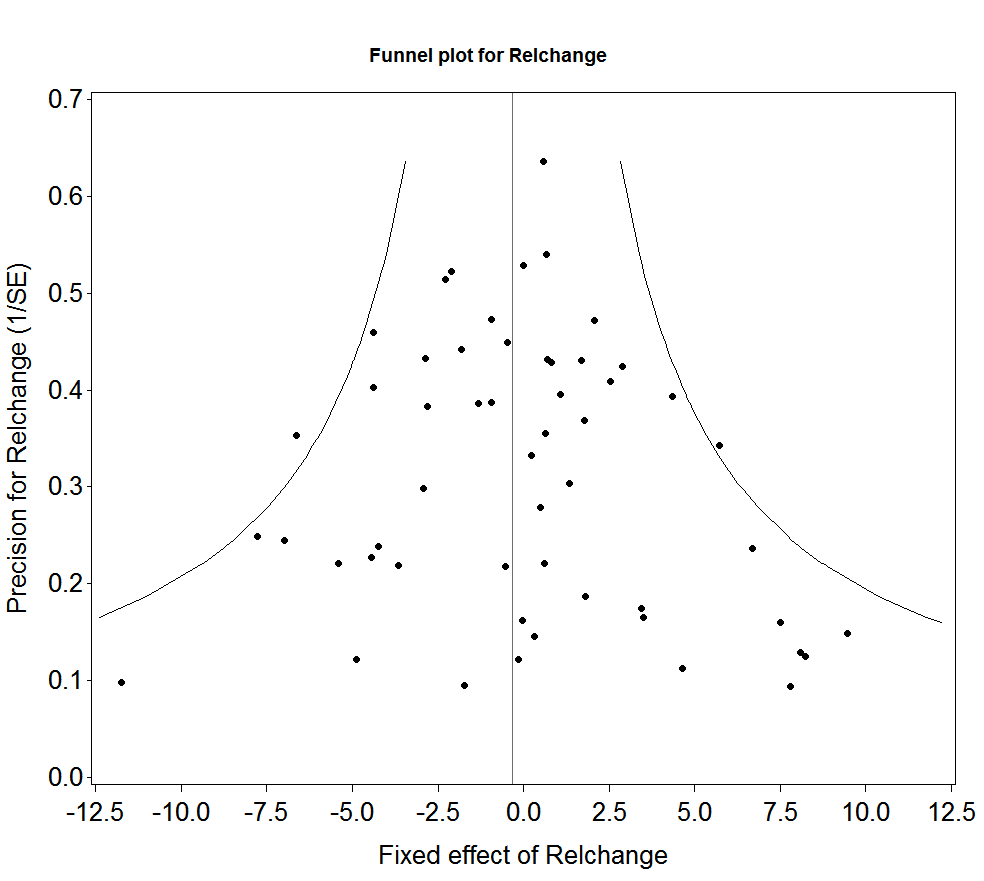


I^2^ = 29.9; P-value = 0.023

Egger test: P-value (intercept) = 0.801

I^2^ = 1.4; P-value = 0.445

Egger test: P-value (intercept) = 0.556

-17.5 -15.0 -12.5 -10.0 -7.5 -5.0 -2.5 0.0 2.5 5.0 7.5 10.0 12.5 15.0 17.5

Precision (1/SE)

Supplemental Figure 3: Funnel plot relative change in non-standardized (left panel) and TC-standardized (right panel) plasma α-tocopherol concentrations
